# The SANAD II study of the effectiveness and cost-effectiveness of levetiracetam, zonisamide, or lamotrigine for newly diagnosed focal epilepsy: an open-label, non-inferiority, multicentre, phase 4, randomised controlled trial

**DOI:** 10.1016/S0140-6736(21)00247-6

**Published:** 2021-04-10

**Authors:** Anthony Marson, Girvan Burnside, Richard Appleton, Dave Smith, John Paul Leach, Graeme Sills, Catrin Tudur-Smith, Catrin Plumpton, Dyfrig A Hughes, Paula Williamson, Gus A Baker, Silviya Balabanova, Claire Taylor, Richard Brown, Dan Hindley, Stephen Howell, Melissa Maguire, Rajiv Mohanraj, Philip E Smith, SANAD II collaborators, Karen Lanyon, Mark Manford, Manali Chitre, Alasdair Parker, Nina Swiderska, Richard Appleton, James Pauling, Adrian Hughes, Rajat Gupta, Sadia Hanif, Mostafa Awadh, Sharmini Ragunathan, Nicola Cable, Paul Cooper, Daniel Hindley, Karl Rakshi, Sophie Molloy, Markus Reuber, Kunle Ayonrinde, Martin Wilson, Satyanarayana Saladi, John Gibb, Lesley-Ann Funston, Damhait Cassidy, Jonathan Boyd, Mal Ratnayaka, Hani Faza, Martin Sadler, Hassan Al-Moasseb, Clare Galtrey, Damien Wren, Anas Olabi, Geraint Fuller, Muhammed Khan, Chetana Kallappa, Ravi Chinthapalli, Baba Aji, Rhys Davies, Kathryn Foster, Nikolas Hitiris, Melissa Maguire, Nahin Hussain, Simon Dowson, Julie Ellison, Basil Sharrack, Vandna Gandhi, Rob Powell, Phil Tittensor, Beatrice Summers, Sastry Shashikiran, Penelope J Dison, Shanika Samarasekera, Doug McCorry, Kathleen White, Kannan Nithi, Martin Richardson, Richard Brown, Rupert Page, David Deekollu, Sean Slaght, Stephen Warriner, Mansoor Ahmed, Abhijit Chaudhuri, Gabriel Chow, Javier Artal, Danute Kucinskiene, Harish Sreenivasa, Singara Velmurugan,, Christos S Zipitis, Brendan McLean, Vaithianathar Lal, Angelous Gregoriou, Paul Maddison, Trevor Pickersgill, Joseph Anderson, Charlotte Lawthom, Stephen Howell, Gabriel Whitlingum, Wojtek Rakowicz, Lucy Kinton, Alisa McLellan, Nitish Vora, Sameer Zuberi, Andrew Kelso, Imelda Hughes, John Martland, Hedley Emsley, Christian de Goede, RP Singh, Carl-Christian Moor, Julia Aram, Rajiv Mohanraj, Kumar Sakthivel, Suresh Nelapatla, Chris Rittey, Ashwin Pinto, John Paul Leach, Hannah Cock, Anna Richardson, Erika Houston, Christopher Cooper, Geoff Lawson, Albert Massarano, Christine Burness, Anthony Marson, Dave Smith, Udo Wieshmann, Indranil Dey, Puthuval Sivakumar, Lap-Kong Yeung, Philip Smith, Hemalata Bentur, Tom Heafield, Anna Mathew, David Smith, Praveen Jauhari

**Affiliations:** a Department of Molecular and Clinical Pharmacology, University of Liverpool, Liverpool, UK; b Department of Health Data Science, University of Liverpool, Liverpool, UK; c Liverpool Clinical Trials Centre, University of Liverpool, Liverpool, UK; d The Roald Dahl EEG Unit, Alder Hey Children's Health Park, Liverpool, UK; e The Walton Centre NHS Foundation Trust, Liverpool, UK; f School of Medicine, University of Glasgow, Glasgow, UK; g Centre for Health Economics and Medicines Evaluation, Bangor University, Bangor, Wales, UK; h Addenbrooke's Hospital NHS Foundation Trust, Cambridge, UK; i Bolton NHS Foundation Trust, Royal Bolton Hospital, Lancashire, UK; j Department of Neurology, Royal Hallamshire Hospital, Sheffield, UK; k School of Medicine, University of Leeds, Leeds, UK; l Salford Royal NHS Foundation Trust, Manchester, UK; m The Alan Richens Epilepsy Unit, University Hospital of Wales, Cardiff, Wales, UK

## Abstract

**Background:**

Levetiracetam and zonisamide are licensed as monotherapy for patients with focal epilepsy, but there is uncertainty as to whether they should be recommended as first-line treatments because of insufficient evidence of clinical effectiveness and cost-effectiveness. We aimed to assess the long-term clinical effectiveness and cost-effectiveness of levetiracetam and zonisamide compared with lamotrigine in people with newly diagnosed focal epilepsy.

**Methods:**

This randomised, open-label, controlled trial compared levetiracetam and zonisamide with lamotrigine as first-line treatment for patients with newly diagnosed focal epilepsy. Adult and paediatric neurology services across the UK recruited participants aged 5 years or older (with no upper age limit) with two or more unprovoked focal seizures. Participants were randomly allocated (1:1:1) using a minimisation programme with a random element utilising factor to receive lamotrigine, levetiracetam, or zonisamide. Participants and investigators were not masked and were aware of treatment allocation. SANAD II was designed to assess non-inferiority of both levetiracetam and zonisamide to lamotrigine for the primary outcome of time to 12-month remission. Anti-seizure medications were taken orally and for participants aged 12 years or older the initial advised maintenance doses were lamotrigine 50 mg (morning) and 100 mg (evening), levetiracetam 500 mg twice per day, and zonisamide 100 mg twice per day. For children aged between 5 and 12 years the initial daily maintenance doses advised were lamotrigine 1·5 mg/kg twice per day, levetiracetam 20 mg/kg twice per day, and zonisamide 2·5 mg/kg twice per day. All participants were included in the intention-to-treat (ITT) analysis. The per-protocol (PP) analysis excluded participants with major protocol deviations and those who were subsequently diagnosed as not having epilepsy. Safety analysis included all participants who received one dose of any study drug. The non-inferiority limit was a hazard ratio (HR) of 1·329, which equates to an absolute difference of 10%. A HR greater than 1 indicated that an event was more likely on lamotrigine. The trial is registered with the ISRCTN registry, 30294119 (EudraCt number: 2012-001884-64).

**Findings:**

990 participants were recruited between May 2, 2013, and June 20, 2017, and followed up for a further 2 years. Patients were randomly assigned to receive lamotrigine (n=330), levetiracetam (n=332), or zonisamide (n=328). The ITT analysis included all participants and the PP analysis included 324 participants randomly assigned to lamotrigine, 320 participants randomly assigned to levetiracetam, and 315 participants randomly assigned to zonisamide. Levetiracetam did not meet the criteria for non-inferiority in the ITT analysis of time to 12-month remission versus lamotrigine (HR 1·18; 97·5% CI 0·95–1·47) but zonisamide did meet the criteria for non-inferiority in the ITT analysis versus lamotrigine (1·03; 0·83–1·28). The PP analysis showed that 12-month remission was superior with lamotrigine than both levetiracetam (HR 1·32 [97·5% CI 1·05 to 1·66]) and zonisamide (HR 1·37 [1·08–1·73]). There were 37 deaths during the trial. Adverse reactions were reported by 108 (33%) participants who started lamotrigine, 144 (44%) participants who started levetiracetam, and 146 (45%) participants who started zonisamide. Lamotrigine was superior in the cost-utility analysis, with a higher net health benefit of 1·403 QALYs (97·5% central range 1·319–1·458) compared with 1·222 (1·110–1·283) for levetiracetam and 1·232 (1·112, 1·307) for zonisamide at a cost-effectiveness threshold of £20 000 per QALY. Cost-effectiveness was based on differences between treatment groups in costs and QALYs.

**Interpretation:**

These findings do not support the use of levetiracetam or zonisamide as first-line treatments for patients with focal epilepsy. Lamotrigine should remain a first-line treatment for patients with focal epilepsy and should be the standard treatment in future trials.

**Funding:**

National Institute for Health Research Health Technology Assessment programme.

**Research in context**

**Evidence before this study**

At the time of the design of this trial (SANAD II) lamotrigine had been identified as a first-line treatment for patients with newly diagnosed focal epilepsy based in part on results of the first SANAD trial (published in 2007), which found lamotrigine to be non-inferior to carbamazepine for seizure remission but significantly less likely to fail as it is better tolerated. Lamotrigine was also found to be more clinically effective than gabapentin or topiramate and was also identified as a cost-effective alternative. Lamotrigine was therefore selected as the standard treatment in SANAD II.

Levetiracetam and zonisamide had been licensed for use as monotherapy in patients with focal epilepsy on the basis of non-inferiority regulatory trials in which they had been compared with carbamazepine. The primary outcome in those trials was 6-month seizure freedom, which is too short a timeframe to assess clinical effectiveness in a long-term condition such as epilepsy. Consequently, the longer term clinical effectiveness and cost-effectiveness of levetiracetam and zonisamide were unknown. This knowledge gap is reflected in the National Institute for Health and Care Excellence epilepsy guidelines, which do not recommend either drug as a first-line treatment. Nonetheless, levetiracetam in particular has been increasingly prescribed in clinical practice on the basis of assumptions about its ease of use, tolerability, and efficacy.

A Cochrane review and individual participant data network meta-analysis that included data from SANAD I found that, compared with carbamazepine, levetiracetam was inferior for time to 12-month remission and no significant difference was found compared with zonisamide. For time to treatment failure, levetiracetam was found to be superior to carbamazepine, but no difference was found between carbamazepine and zonisamide.

**Added value of this study**

To the best of our knowledge, this study is the first randomised controlled trial to assess the longer term clinical effectiveness and cost-effectiveness of levetiracetam and zonisamide as first-line treatments for patients with newly diagnosed focal epilepsy. The study is pragmatic in design and recruited a cohort of participants older than 5 years from routine UK National Health Service clinics, and the results are relevant to every day clinical practice.

Levetiracetam did not meet our definition of non-inferiority for time to 12-month remission compared with lamotrigine, and it was inferior for time to treatment failure. Zonisamide did meet our definition of non-inferiority for time to 12-month remission but it too was inferior for time to treatment failure. Neither levetiracetam or zonisamide were found to be cost-effective alternatives.

**Implications of all the available evidence**

For people with focal epilepsy, there is evidence that both levetiracetam and zonisamide have efficacy when used as monotherapy. However, the evidence does not support their use as first-line treatments. Our results support the continued use of lamorigine as first-line treatment for patients with focal epilepsy and its use as a standard comparator in future comparative trials.

## Introduction

Epilepsy is a common condition with a prevalence of 0·5–1% and lifetime incidence of up to 5%.^1^, ^2^ Epilepsy is a complex disorder with many seizure types and causes. It is uniquely stigmatising and negatively affects quality of life (QOL), education, and employment prospects.^3^, ^4^ Anti-seizure medications are the mainstay of treatment and approximately 70% of people with epilepsy will achieve a remission from seizures, half of whom may be able to stop treatment without a seizure recurrence.

Around two-thirds of people with epilepsy have focal epilepsy; seizures that start in a neuronal network limited to one cerebral hemisphere. Seizure types include focal aware seizures (previously called simple partial seizures), focal seizures with impaired awareness (previously called complex partial seizures), and focal to bilateral tonic-clonic seizures (previously called secondarily generalised tonic clonic seizures).^5^, ^6^, ^7^ Focal epilepsy can start at any age, and the incidence distribution is U shaped, with higher incidence in young and elderly people. Because of ageing populations, in many countries the incidence of focal epilepsy is higher in older people than in younger people.^2^

Although focal epilepsies can be classified according to site of seizure onset and cause, there is no evidence to suggest that one syndrome or cause responds better to one specific treatment than to another.^8^ The drug management is therefore generally similar whatever the cause or syndrome. Guidelines typically recommend lamotrigine or carbamazepine as first-line treatment,^9^ in part informed by the first standard and new antiepileptic drug trial (SANAD I),^10^ which identified lamotrigine as non-inferior to carbamazepine for time to 12-month remission and superior to carbamazepine, gabapentin, oxcarbazepine, and topiramate for time to treatment failure.

Since the publication of SANAD I, several newer treatments have become available, including levetiracetam and zonisamide. Levetiracetam is a commonly prescribed anti-seizure medication with evidence of efficacy as monotherapy in patients with focal epilepsy based on finding non-inferiority compared with carbamazepine for 6-month seizure remission and similar tolerability in a regulatory trial^11^ that did not assess longer term effectiveness. A second open-label trial^12^ compared levetiracetam with physicians' choice of carbamazepine or valproate, and it showed no significant difference between carbamazepine and levetiracetam for time to first seizure and time to treatment failure. However, that trial^12^ had a maximum follow-up of 12 months and therefore could not assess longer term outcomes needed to inform policy. In the 2012 NICE epilepsy guideline,^9^ levetiracetam was not recommended as a first-line treatment on the basis of an analysis indicating that it was not cost-effective. However, levetiracetam has since become available in generic form and is widely prescribed.

Zonisamide has been available for many years in Japan^13^ and southeast Asia. Its licence for use as a monotherapy is also based on a regulatory study^14^ showing non-inferiority to carbamazepine for 6-month seizure remission rates. The longer term comparative clinical and cost-effectiveness of zonisamide is unknown.

The aim of SANAD II was to assess the longer term clinical effectiveness and cost-effectiveness of levetiracetam and zonisamide compared with lamotrigine in people with newly diagnosed focal epilepsy.

## Methods

### Study design and participants

SANAD-II was a phase 4, multicentre, non-inferiority, open-label, randomised controlled trial that was run in 65 UK National Health Service (NHS) adult neurology and paediatric services. Participants were eligible for recruitment if they were aged 5 years or older (there was no upper age limit), had a history of at least two unprovoked epileptic seizures requiring anti-seizure medication, their clinical diagnosis was one of focal epilepsy with or without an electroencephalogram, and they had never been treated with an anti-seizure medication except for emergency treatment in the previous 2 weeks. Exclusion criteria included patients with provoked or acute symptomatic seizures only, patients currently taking anti-seizure medication, and known progressive neurological disease (eg, brain tumour). Epileptic seizures and syndromes were classified according to International League Against Epilepsy classifications.^5^, ^6^ SANAD II was granted ethics approval from the North West-Liverpool East Research Ethics Committee (Ref 12/NW/0361) on June 7, 2012.

### Randomisation and masking

After providing consent, participants were randomly allocated (1:1:1) to receive either lamotrigine, levetiracetam, or zonisamide. We used a secure, centrally controlled, 24-h web-based facility to implement a minimisation program with random element utilising factors, which were not made known to reduce the risk of predicting allocation. These factors were centre, sex (ie, male or female), and number of previous seizures (ie, 2, 3–5, 6+). Recruiting clinicians were required to initiate trial treatment within 7 days of randomisation. Participants and investigators were not masked and were all aware of treatment allocation.

### Procedures

SANAD II was an open-label trial. Trial treatments were prescribed as per routine NHS practice and dispensed by hospital and community pharmacies, and clinicians prescribed the formulation they considered most appropriate. The trial protocol provided guidance on initial drug titration and maintenance doses based on routine practice, although clinicians were able to tailor these as appropriate. All anti-seizure medications were taken orally. For participants aged 12 years or more, the initial advised maintenance doses were lamotrigine (50 mg in the morning and 100 mg in the evening), levetiracetam (500 mg twice per day), and zonisamide (100 mg twice per day). For children aged 5–12 years, the initial daily maintenance doses advised were lamotrigine (1·5 mg/kg twice per day), levetiracetam (20 mg/kg twice per day), and zonisamide (2·5 mg/kg twice per day). Subsequent dose and treatment changes at follow-up visits were done following routine clinical practice according to treatment response and adverse effects.

We aimed to complete recruitment over a 4·5 year period but a 12-month extension was required to meet the sample size target, after which the trial cohort was followed up for a further 2 years, allowing a minimum follow up of 2 years and maximum of 7·5 years. Patients were followed up according to clinical need, and minimum trial visits were expected at 3, 6, and 12 months, and annually thereafter. At visits, data were collected for seizure type and frequency, anti-seizure medication, and adverse reactions. Participants continued in follow-up whether they were still taking their allocated treatment or not. For participants lost to hospital follow-up, outcome data were sought from their general practitioner.

For adults, QOL outcomes were assessed using subscales of the quality of life in newly diagnosed epilepsy battery (NEWQOL) and the Impact of Epilepsy Scale.^15^ For children and adolescents aged <16 years, QOL assessment involved both patient and parent-based measures: children aged 8–15 years completed a generic health status measure validated for use in epilepsy, the KINDL;^16^ and the epilepsy impact and attitude to epilepsy subscales of the Quality of Life in Epilepsy Inventory for Adolescents (QOLIE-AD).^17^ Parents of all children completed proxy QOL questionnaires. QOL questionnaires were completed at baseline and annually thereafter. Adults and parents also completed a subset of QOL measures at 3 months and 6 months.

Adult and adolescent participants were asked to complete the EQ-5D-3L and the EuroQol visual analogue scale (EQ-VAS); participants aged 8–15 completed the EQ-VAS and self-reported youth EQ-5D-3L-Y, or if not available, proxy EQ-5D-3L, completed by a parent or carer. For participants aged 5–7 years, only proxy questionnaires were administered. Participants' resource-use associated with secondary care (ie, inpatient, outpatient, and accident and emergency), other health-care and social services (ie, primary care and community services), and medicines were measured using routine hospital episode statistics, resource-use questionnaires,^18^ and case report form records. Resource-use was valued in monetary terms (measured in pounds sterling using national unit costs for 2019–20) using national unit costs.^19^, ^20^, ^21^, ^22^

### Outcomes

The primary outcome was time to 12-month remission from seizures, calculated as days from randomisation to the first date at which a period of 12 months had elapsed without the patient having any seizures. The secondary seizure outcomes were time to 24-month remission and time to first seizure. There were three secondary outcomes for treatment failure: (1) time to treatment failure overall, defined as days from randomisation to a decision to withdraw the randomised drug, or to add a new anti-seizure medication because of inadequate seizure control or unacceptable adverse reactions; (2) time to treatment failure due to inadequate seizure control; and (3) time to treatment failure due to unacceptable adverse reactions. The other secondary outcomes were adverse reactions, QOL, and health economic outcomes based on incremental costs and quality-adjusted life-years (QALYs).

### Statistical analysis

SANAD-II was designed to detect non-inferiority of both levetiracetam and zonisamide compared with lamotrigine for the primary outcome of time to 12-month remission. The International League Against Epilepsy Commission on Antiepileptic Drugs defined limits of equivalence of ±10% for the primary outcome in antiepileptic drug monotherapy studies.^23^ Calculations were informed by the SANAD I study,^9^ which estimated the 12-month remission-free probability (at 24 months) of 0·43 (and an exponential hazard rate of 0·0352) for lamotrigine. Two primary comparisons were of interest (levetiracetam *vs* lamotrigine, and zonisamide *vs* lamotrigine), therefore the one-sided significance level was divided by two (one-side alpha of 0·0125). Assuming a 10% absolute difference in survival probability, the non-inferiority margin on the hazard ratio (HR) scale was ln(0·43)/ln(0·53)=1·329. Therefore, assuming a HR of 1 and 80% power with a one-sided alpha of 0·025, 330 patients were required in each of three treatment groups, allowing for 5% losses to follow-up, as occurred in SANAD I (990 total patients).

Primary analyses were done on an intention-to-treat (ITT) basis. We used a 0·025 level of significance and 97·5% CIs for analysis of the primary outcome because of the two prespecified comparisons. All secondary outcomes were analysed using a 0·05 level of significance and 95% CIs. The statistical and health economic analysis plans were developed before doing the final analyses and are available in appendix 1 and appendix 2. Analyses were done using SAS software (version 9.4; SAS Institute, Cary, NC, USA). Completeness of follow-up statistics were calculated as the total number of days follow-up for all participants as a percentage of the total potential number of days follow-up.^24^

Time to event outcomes were summarised by Kaplan-Meier curves for each treatment group and Cox proportional hazards regression models explored using two different models: (1) the primary analysis, including the treatment effect only; (2) including the treatment together with gender (ie, male or female), number of seizures before randomisation (ie, 2, 3–5, 6+); and random effects for centre. The assumption of proportional hazards was investigated by examining Schoenfeld residual plots and incorporating time-dependent covariates in all models. If the assumption of proportional hazards was not valid, an additional Cox model with time-dependent covariates was used. For the primary outcome (ie, 12-month remission) non-inferiority hypothesis, we present 97·5% CIs, in which the upper limit of the 97·5% CI needed to be less than 1·329 to conclude non-inferiority. All other treatment effects are presented as a HR with a two-sided 95% CI of lamotrigine compared with levetiracetam or zonisamide.

A per-protocol (PP) analysis of the primary outcome was also done using the Fine and Gray model,^18^ with treatment failure included as a competing risk, and censoring participants with drug failure before achieving remission. This analysis excluded participants with major protocol deviations, participants subsequently given an alternative diagnosis to epilepsy, and participants who did not receive the drug at all.

For time to treatment failure, a competing risks analysis, using the Fine and Gray model,^18^ was done to assess the two main reasons for treatment failure (ie, inadequate seizure control and unacceptable adverse reactions).^19^ Cumulative incidence curves are presented for each treatment group.

The difference in QOL measures between treatment groups was estimated for each population (ie, children, adults, and parent-carers) and for each outcome applicable within that population by fitting a repeated measures random effects model with a baseline QOL variable, treatment group, and time in days using spatial-power covariance structure for repeated measures and unstructured covariance for the random effect. QALYs were calculated on the basis of the area under the curve of utility data measured using the EuroQol 5-dimension 3-level (EQ-5D-3L) questionnaire, and applying the UK tariff scores.^17^

Analysis sets for the summary of adverse reactions included all patients who received any dose of a study drug. All adverse reactions and serious adverse reactions were coded using the MedDRA dictionary. The number (and percentage) of patients experiencing each reaction and the number (and percentage) of occurrences of each reaction are presented with no formal statistical testing undertaken.

Interim monitoring was done by an independent data safety and monitoring committee, meeting approximately annually. This process included analyses of the primary outcome and five of the secondary outcomes (all using the Haybittle-Peto approach).

QALYs were calculated on the basis of the area under the curve of utility data measured using the EuroQol 5-dimension 3-level (EQ-5D-3L) questionnaire, and applying the UK tariff scores.^17^

### Health economic evaluation

The economic analysis (appendix 2) adopted the costing perspective of the NHS and personal social services and was done using data up to 24-months of follow-up and in accordance with the NICE guidelines for the methods of technology appraisal.^20^ Missing cost and QALY data were imputed using multiple imputation with chained equations.^21^ Based on the imputed data, differences between treatment groups in total costs and QALYs were compared with reference to bootstrapped central ranges, based on 10 000 replications. In the base-case analysis, total costs and QALYs (year 2 discounted at 3·5%) were adjusted using linear regressions^25^ for treatment allocation, baseline costs or utility, age, sex, and epilepsy classification, with centre as random effects. Incremental costs and QALYs were estimated to identify dominance, and calculate the incremental net health benefit as the difference in QALYs between treatments, minus the difference in costs multiplied by the cost-effectiveness threshold (ie, £20 000 per QALY).^20^ The joint uncertainty in incremental costs and QALYs were expressed in terms of the probability of each treatment being cost-effective at this threshold. Sensitivity analyses comprised alternative discount rates, use of complete cases, PP cohort, QALYs based on the NEWQOL-6D^26^ and EQ-VAS, and were based on unadjusted analyses. A subgroup analysis considered cost-effectiveness in children, adults, and adolescents aged 16 years or more at the point of random assignment.

### Role of the funding source

The funder of the study had no role in study design, data collection, data analysis, data interpretation, or writing of the report.

## Results

**Figure 1:**
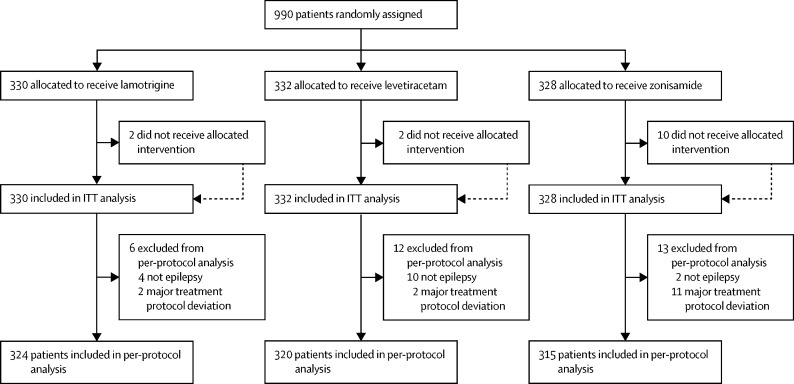
Trial profile Data on non-randomised patients were not collected. ITT=intention-to-treat.

The first participant was randomly assigned on May 2, 2013, and the last participant was randomly assigned on June 20, 2017, after which every effort was made to follow the trial cohort for a further 2 years; the last participant follow-up visit was on October 17, 2019. 65 UK centres recruited between one and 130 patients each, and randomly assigned a total of 990 participants, 330 to start treatment with lamotrigine, 332 to start treatment with levetiracetam, and 328 to start treatment with zonisamide (figure 1). Baseline characteristics were well balanced across treatment groups (table 1). The mean age of participants was 39·3 years (SD 21·2), and 177 (17·9%) of 990 participants were younger than 18 years.

**Table 1:** Baseline characteristics of the patients

	**Lamotrigine (n=330)**	**Levetiracetam (n=332)**	**Zonisamide (n=328)**	**Total (n=990)**
**Age, years**				
Mean (SD; range)	40·1 (21·7; 5·1–91·9)	37·8 (20·1; 5·0–87·6)	39·9 (21·6; 5·0–89·1)	39·3 (21·2; 5·0–91·9)
**Gender**				
Male	186 (56%)	190 (57%)	185 (56%)	561 (57%)
Female	144 (44%)	142 (43%)	143 (44%)	429 (43%)
**History**				
Learning disability	15 (5%)	16 (5%)	14 (4%)	45 (5%)
Febrile convulsions	10 (3%)	19 (6%)	15 (5%)	44 (4%)
Acute symptomatic seizures	6 (2%)	9 (3%)	4 (1%)	19 (2%)
History of epilepsy in primary relatives	32 (10%)	35 (11%)	40 (12%)	107 (11%)
Neurological deficit	12 (4%)	20 (6%)	12 (4%)	44 (4%)
**Previous or current neurological disorder**				
Any previous or current neurological disorder	57 (17%)	55 (17%)	51 (16%)	163 (16%)
Stroke or cerebrovascular	17 (5%)	16 (5%)	14 (4%)	47 (5%)
Cerebral haemorrhage	2 (1%)	5 (2%)	7 (2%)	14 (1%)
Intracranial surgery	4 (1%)	6 (2%)	10 (3%)	20 (2%)
Head injury	4 (1%)	7 (2%)	7 (2%)	18 (2%)
Meningitis or encephalitis	6 (2%)	5 (2%)	6 (2%)	17 (2%)
Cortical dysplasia or developmental anomaly	1 (<1%)	3 (1%)	0	4 (<1%)
Other	27 (8%)	24 (7%)	18 (5%)	69 (7%)
**Epilepsy syndrome**				
Self-limiting childhood epilepsy with centro-temporal spikes	9 (3%)	15 (5%)	10 (3%)	34 (3%)
Childhood epilepsy with occipital paroxysms	0	1 (<1%)	0	1 (<1%)
Temporal lobe	134 (41%)	110 (33%)	111 (34%)	355 (36%)
Frontal lobe	21 (6%)	21 (6%)	20 (6%)	62 (6%)
Parietal lobe	7 (2%)	8 (2%)	5 (2%)	20 (2%)
Occipital lobe	7 (2%)	12 (4%)	2 (1%)	21 (2%)
Focal epilepsy localisation not specified	152 (46%)	165 (50%)	182 (55%)	499 (50%)
Other epilepsy syndrome	3 (1%)	1 (<1%)	1 (<1%)	5 (1%)
**Seizure history (median, IQR)**				
Total number of seizures reported	6 (3–29)	6 (3–22)	6 (3–23)	6 (3–24)
Days since first seizure	333 (110–1090)	318 (119–985)	328 (120–1097)	327 (114–1035)
Days since most recent seizure	13 (3–41)	13 (3–35)	11 (3–34)	13 (3–36)

Data are n (%), median (IQR), or mean (SD).

There was a predominance of male participants (561 participants; 57%); 45 (5%) had a learning disability, 163 (16%) had a previous or current neurological disorder, 107 (11%) had a first degree relative with epilepsy, 44 (4%) had a history of febrile convulsions. Moreover, 355 (36%) participants were classified with temporal lobe epilepsy, 62 (6%) had frontal lobe epilepsy, 21 (2%) had occipital lobe epilepsy, 20 (2%) had parietal lobe epilepsy, and 499 (50%) had focal epilepsy localisation not specified. The median number of seizures before randomisation was 6 (IQR 3–24) and participants were randomly assigned a median of 13 days (3–36) after their most recent seizure.

The median (IQR) number of days of follow-up was 462·5 (365–777) for lamotrigine, 449·5 (365–824) for levetiracetam, and 447 (365–730) for zonisamide, with completeness of follow-up statistics for the primary outcome of 77% for lamotrigine, 78% for levetiracetam, and 76% for zonisamide.

**Figure 2:**
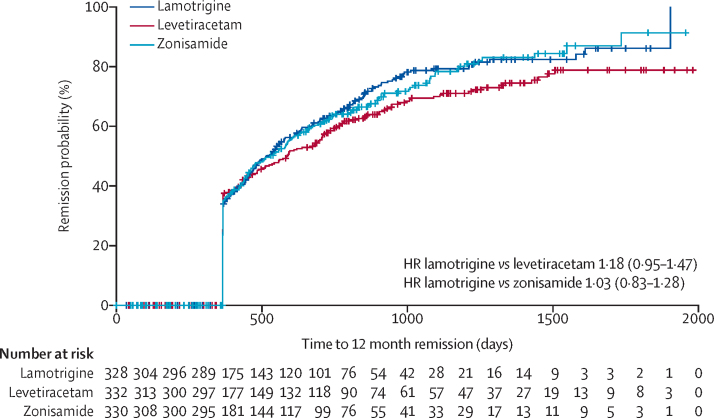
Kaplan-Meier plot of time to 12-month remission: lamotrigine versus levetiracetam and lamotrigine versus zonisamide, intention-to-treat analysis HR=hazard ratio.

Levetiracetam did not meet our definition of non-inferiority to lamotrigine as the 97·5% CI for the HR (1·18 [97·5% CI 0·95 to 1·47] unadjusted, 1·13 [0·91 to 1·41] adjusted]) included the pre-defined non-inferiority margin of 1·329. Consequently, we were unable to exclude the possibility of an important clinical difference between levetiracetam and lamotrigine. Zonisamide did meet our definition of non-inferiority to lamotrigine HR (1·03 [0·83 to 1·28] unadjusted, 1·01 [0·81 to 1·26] adjusted). There was no evidence of violation of the assumption of proportional hazards (p=0·90). Annual differences in annual 12-month remission probabilities, for example, that at 2 years follow-up we estimated that 5% fewer participants had a remission on levetiracetam compared with lamotrigine (95% CI −13 to 3) and 1% fewer on zonisamide compared with lamotrigine (–9 to 7), are shown in appendix 3 (p 2). The Kaplan-Meier estimates of the median time to achieve 12-month remission were 516 days (95% CI 457–577) for lamotrigine, 588 days (472–706) for levetiracetam, and 530 days (453–601) for zonisamide (figure 2).

The PP analyses (using the Fine and Gray model) for time to 12-month remission (appendix 3 p 3) excluded patients with major protocol deviations (15 participants; 1·5%) and patients later diagnosed as not meeting the diagnosis of epilepsy (16 participants; 1·6%), and accounted for treatment failures before achieving 12-month remission (ie, 78 [24%] participants in the lamotrigine group, 177 [35%] participants in the levetiracetam group, and 127 [39%] participants in the zonisamide group) in a competing risks analysis. Results from the per-protocol analysis showed that the 12-month remission was superior with lamotrigine than both with levetiracetam (HR 1·32 [97·5% CI 1·05 to 1·66]) and with zonisamide (HR 1·37 [1·08–1·73]); although this trial was powered for non-inferiority, it was also designed to show superiority.

**Figure 3:**
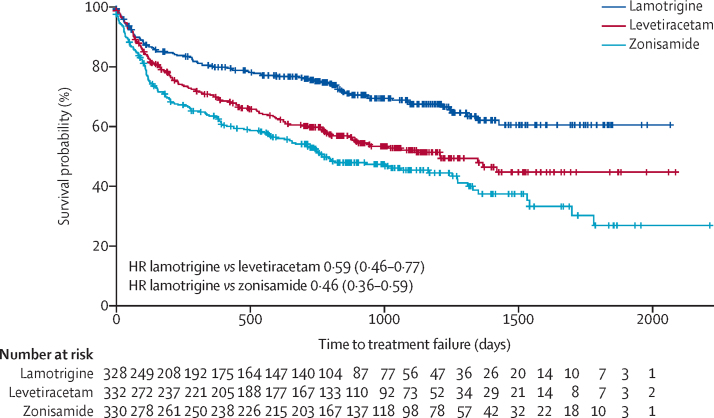
Kaplan-Meier plot of time to treatment failure: lamotrigine versus levetiracetam and lamotrigine versus zonisamide HR=hazard ratio.

No significant difference was found in time to 24-month remission (using ITT analysis) for lamotrigine versus levetiracetam (HR 1·04 [95% CI 0·81–1·33]) or for lamotrigine versus zonisamide (0·96 [0·75–1·23]) (appendix 3 p 4). No significant difference was found in time to first seizure (using ITT analysis) for lamotrigine versus levetiracetam (HR 1·07 [95% CI 0·89–1·29]) or lamotrigine versus zonisamide (1·04 [0·86–1·25]; appendix 3 p 5). The analysis of overall time to treatment failure for any reason (figure 3) found lamotrigine to be significantly less likely to fail than levetiracetam (0·60 [0·46–0·77]) or than zonisamide (0·46 [0·36–0·60]), with no evidence against an assumption of proportional hazards (p=0·77). Annual treatment failure rates and differences in failure rates between lamotrigine and both levetiracetam and zonisamide are shown in appendix 3 (p 6). Compared with lamotrigine, there were 16% (95% CI 9–23) more treatment failures on levetiracetam and 23% (15–30) more treatment failures on zonisamide at 2 years. The doses taken at treatment failure or last follow-up and indicate that reasonable dose ranges were tried before deciding failure had occurred (appendix 3 p 7).

The competing risks analysis (appendix 3 p 8) shows that levetiracetam was significantly more likely to fail than lamotrigine because of adverse reactions (HR 0·53 [95% CI 0·35–0·79]) but not inadequate seizure control (0·67 [0·45–1·01]). Similarly, zonisamide was significantly more likely to fail than lamotrigine because of adverse reactions (0·37 [0·25–0·55]) but not because of inadequate seizure control (0·76 [0·50–1·15]).

SANAD II recorded data for adverse reactions, which were adverse events judged by the treating clinicians to be possibly, probably, or definitely caused by anti-seizure medication. Table 2 provides an ITT (by treatment policy) summary of adverse reactions according to the MEDRA system organ classification. Participants were included in the safety analysis if they took at least one dose of their allocated treatment.

**Table 2:** Adverse reactions by MedDRA system organ classification

	**Number of events**			**Number of patients (%)**		
	Lamotrigine	Levetiracetam	Zonisamide	Lamotrigine (n=328)	Levetiracetam (n=330)	Zonisamide (n=324)
Psychiatric disorders	58	147	103	43 (13%)	98 (30%)	73 (23%)
Nervous system disorders	88	81	85	53 (16%)	55 (17%)	60 (19%)
General disorders and administration site conditions*	23	37	44	17 (5%)	32 (10%)	39 (12%)
Gastrointestinal disorders	30	29	35	25 (8%)	22 (7%)	26 (8%)
Skin and subcutaneous tissue disorders†	29	14	28	24 (7%)	12 (4%)	21 (7%)
Investigations	6	11	16	6 (2%)	11 (3%)	16 (5%)
Metabolism and nutrition disorders	4	2	17	3 (1%)	2 (1%)	16 (5%)
Musculoskeletal and connective tissue disorders	5	1	8	5 (2%)	1 (<1%)	7 (2%)
Eye disorders	1	1	5	1 (<1%)	1 (<1%)	5 (2%)
Renal and urinary disorders	1	0	6	1 (<1%)	0	5 (2%)
Cardiac disorders	2	2	1	2 (<1%)	2 (1%)	1 (<1%)
Respiratory, thoracic, and mediastinal disorders	1	1	2	1 (<1%)	1 (<1%)	2 (1%)
Injury, poisoning, and procedural complications	2	0	0	2 (1%)	0	0
Ear and labyrinth disorders	0	1	0	0	1 (<1%)	0
Endocrine disorders	0	1	0	0	1 (<1%)	0
Pregnancy, puerperium, and perinatal conditions	0	0	1	0	0	1 (<1%)
Vascular disorders	1	0	0	1 (<1%)	0	0
Total events and patients with at least one adverse reaction	251	328	351	108 (33%)	144 (44%)	146 (45%)

* 88 (85%) of 104 adverse reactions in this category were fatigue.

† 42 events were rash: 22 events in the lamotrigine group, eight events in the levetiracetam group, and 12 events in the zonisamide group. Two of these were categorised as severe: one dermatitis allergic in the lamotrigine group and one Stevens Johnson syndrome in the levetiracetam group.

There were 251 adverse reactions experienced by 108 (33%) participants who initiated treatment with lamotrigine, 328 adverse reactions experienced by 144 (44%) participants who initiated treatment with levetiracetam, and 351 adverse reactions in 146 (45%) participants who initiated treatment with zonisamide. The main difference in adverse reaction profiles was in reporting of psychiatric symptoms: 43 (13%) participants who initiated lamotrigine, 98 (30%) participants who initiated levetiracetam, and 73 (23%) participants who initiated zonisamide.

Seven events were classified as a serious adverse reaction in two participants who initiated lamotrigine, one participant who initiated levetiracetam, four participants who initiated zonisamide, and none were suspected unexpected serious adverse reactions. There were 37 deaths during the trial; 15 in participants who initiated lamotrigine (four possibly seizure related), 12 in participants who initiated levetiracetam (two possibly seizure related), and ten in participants who initiated zonisamide (two possibly seizure related).

There were 11 pregnancies in 11 women who initiated treatment with lamotrigine (ten with normal postnatal examination of the baby and one with minor malformations); six pregnancies in five women who initiated levetiracetam (five women with normal postnatal examination of the baby and one termination); 17 pregnancies in 14 women who initiated treatment with zonisamide; eight with normal postnatal examination of the baby; eight miscarriages (in five women) and one termination.

493 (49·8%) of participants returned QOL questionnaires at baseline and at least one other timepoint during follow-up. For participants who provided data for QOL, the mean follow-up time was 934 days (SD 519) and the maximum was 2289 days. A comparison of participants who did and did not return questionnaires showed a similar proportion of male and female participants and a similar proportion with learning disability and neurological deficits, although participants who returned the questionnaires were a mean of 10 years older (appendix 3 p 9).

Overall for adults, lamotrigine was associated with a better profile on self-reported measures than levetiracetam or zonisamide. A comparison of the treatment effects (appendix 3 p 10) showed negative treatment effects for levetiracetam compared with lamotrigine for patient reported anxiety, depression, stigma, epilepsy impact, and overall QOL. Compared with lamotrigine, zonisamide had a negative treatment effect for depression, epilepsy impact, and overall QOL.

Data for hospital episode statistics were available for 772 participants, and self-reported resource-use data were available for 550 participants at 3 months, 527 participants at 6 months, 465 participants at 12 months, and 398 participants at 24 months. Most of the costs were related to hospital outpatient clinic attendance and admitted care (appendix 3 p 11). In the adjusted, base-case analysis, total costs were £4042 (97·5% CR 3626–4983) for lamotrigine, £5104 (4450–6141) for levetiracetam, and £5400 (4659–6770) for zonisamide (table 3). EQ-5D utilities were available for 616 participants at baseline, and they were calculated for 422 participants at 12 months and for 319 participants at 24 months. Lamotrigine was associated with 1·605 QALYs (97·5% CR 1·547–1·651) in the base-case analysis, compared with 1·474 QALYs (1·393–1·523) with levetiracetam, and 1·502 QALYs (1·418–1·566) with zonisamide. At a cost-effectiveness threshold of £20 000 per QALY, net health benefits were higher, at 1·403 QALYs (1·319–1·458) for lamotrigine, than for levetiracetam 1·222 (1·110–1·283), and for zonisamide 1·232 (1·112–1·307). Lamotrigine therefore dominated both levetiracetam and zonisamide in the base-case analysis, and it had a probability of 0·999 of being cost-effective at this threshold. Lamotrigine also dominated levetiracetam and zonisamide across all sensitivity analyses (with the exception of the analysis of complete cases) and in the subgroup analysis of adults (table 3). However, levetiracetam had the highest net health benefit in participants younger than 16 years. Sensitivity analyses are provided in appendix 4 (pp 29–32).

**Table 3:** Results of the adjusted base-case and subgroup analyses

	**Lamotrigine**	**Levetiracetam**	**Zonisamide**
**Base-case all participants (n=990)**			
Total costs (£)	4042 (3626–4983)	5104 (4450–6141)	5400 (4659–6770)
QALYs	1·605 (1·547–1·651)	1·474 (1·393–1·523)	1·502 (1·418–1·566)
Net health benefit at £20 000 per QALY (QALYs)	1·403 (1·319–1·458)	1·222 (1·110–1·283)	1·232 (1·112–1·307)
**Children aged <16 years (n=155)**			
Total costs (£)	5076 (3815–7219)	4972 (3739–6840)	4638 (3826–6974)
QALYs	1·551 (1·432–1·638)	1·556 (1·397–1·618)	1·508 (1·381–1·610)
Net health benefit at £20 000 per QALY (QALYs)	1·297 (1·127–1·412)	1·307 (1·097–1·394)	1·277 (1·068–1·390)
**Adults and adolescents aged ≥16 years (n=835)**			
Total costs (£)	3844 (3379–4478)	5178 (4435–6223)	5509 (4610–6866)
QALYs	1·612 (1·554–1·661)	1·466 (1·381–1·518)	1·508 (1·412–1·569)
Net health benefit at £20 000 per QALY (QALYs)	1·420 (1·346–1·475)	1·207 (1·095–1·280)	1·227 (1·101–1·320)

Data are mean (97·5% central range). QALY=quality-adjusted life-years.

## Discussion

Our results indicate that lamotrigine should remain a first-line standard treatment for patients with focal epilepsy and that neither levetiracetam or zonisamide should be used routinely as first-line anti-seizure medications. This pragmatic multicentre randomised open-label trial was powered to assess non-inferiority of levetiracetam and zonisamide compared with the standard treatment lamotrigine in patients with newly diagnosed focal epilepsy. Zonisamide met our definition of non-inferiority for the primary outcome time to 12-month remission (ITT analysis) compared with lamotrigine but levetiracetam did not, as the possibility of a clinically important differences could not be excluded. No significant difference was found between lamotrigine and zonisamide for time to 24-month remission and time to first seizure. However, levetiracetam and zonisamide were significantly more likely to fail than lamotrigine and a competing risk analysis indicated that this treatment failure was due mainly to adverse reactions associated with levetiracetam and zonisamide. The PP analysis of time to 12-month remission, which accounted for treatment failure, found lamotrigine to be superior to both levetiracetam and zonisamide.

Initiating treatment with lamotrigine was associated with fewer adverse reactions than levetiracetam or zonisamide and there were more psychiatric adverse reactions associated with levetiracetam and zonisamide. Although there were 37 deaths, there was no indication of a higher death rate with a particular drug, which is reassuring given the concerns reported by the US Food and Drug Association, including cardiac rhythm and conduction abnormalities.^27^ Also, there were more pregnancies and miscarriages for patients who initiated zonisamide, but the numbers are too small to draw any conclusions.

QOL analyses found that, compared with lamotrigine, levetiracetam and zonisamide were associated with worse overall patient reported QOL and depression. In addition, levetiracetam was worse for patient reported anxiety, depression, and stigma.

The cost utility analysis found that neither levetiracetam or zonisamide were cost-effective compared with lamotrigine at thresholds of cost-effectiveness operating in the NHS in the UK. This finding was consistent in sensitivity analyses, and for the subgroup analysis of adults. However, levetiracetam appeared most cost-effective in children who were younger than 16 years, although these results are limited by the small sample size and were principally due to a single participant who had an atypical medical journey.

This study has several limitations. Data for the occurrence of seizures were collected using seizure diaries and reports at clinic visits. It is therefore possible that seizures were missed or not reported, which might have influenced decisions about dose and treatment changes, treatment failure, and reporting of adverse reactions. Only 177 (17·9%) of patients recruited were younger than 18 years, limiting the applicability of our results to children. The most likely explanation for the small number of patients from this age group was because of lack of experience with zonisamide among paediatricians as it is not currently licensed as monotherapy in children. It is possible that the maintenance doses chosen introduced a systematic bias, but the similar time to first seizure rates for lamotrigine, levetiracetam, and zonisamide provide assurance against this possibility, as well as concerns that the slower titration rate required for lamotrigine could expose individuals to a risk of early seizure recurrence. It is also important to acknowledge that there are no data from randomised trials to inform the choice of initial maintenance dose of lamotrigine, levetiracetam, zonisamide, or most other anti-seizure medications, and that in SANAD II, clinicians chose and adjusted doses according to their usual practice. There was also a low return rate for QOL questionnaires and although the rates of return were not unusually low for postal questionnaires, this factor will have diminished our ability to identify differences in QOL between groups. A further limitation of our study was that other newer anti-epileptic drugs were not assessed, in particular lacosamide, which is now licensed as monotherapy for both adults and children, and perampanel, which is only licenced as an adjunctive therapy.

The economic analysis in our study was also limited by the poor return of participants' questionnaires, the inclusion of free-text questions within the resource-use questionnaire, and the assumption that unanswered questions implied no use of resources. However, these limitations were largely mitigated by more complete data for hospital episode statistics given that hospital costs were the main cost driver and were associated with the more complex recall requirement. The effect of missing QALY data was lessened because of the area under the curve methodology, in which QALYs could be calculated provided two or more EQ-5D questionnaires had been returned. Our use of the EQ-5D-3L-Y and proxy version of the EQ-5D-3L was limited by the unavailability of appropriate value sets and by having to apply the adult tariff for estimating utilities. These factors represent a weakness in many economic evaluations of interventions in paediatric populations,^22^ although a valuation of children's EQ-5D-3L-Y health states is in development.^28^

These results should be interpreted in context with previous studies, although most anti-seizure monotherapy randomised controlled trials have been done to meet regulatory requirements^29^, ^30^ and fail to provide evidence about longer term clinical effectiveness. SANAD I identified lamotrigine as a first-line treatment as it was non-inferior to carbamazepine for time to 12-month remission and superior to carbamazepine, gabapentin, oxcarbazepine, and topiramate for time to treatment failure.^10^ An individual patient data network meta-analysis,^31^ which included data from SANAD I and combined direct and indirect comparisons, used carbamazepine as the standard treatment comparator for patients with focal epilepsy. Results indicated that levetiracetam was inferior to carbamazepine for time to 12-month remission and no significant difference was found for carbamazepine compared with gabapentin, lamotrigine, phenobarbital, phenytoin, oxcarbazepine, valproate, or zonisamide. For time to treatment failure, lamotrigine and levetiracetam were superior to carbamazepine, phenobarbital was inferior to carbamazepine, and no difference was found between carbamazepine and the other assessed treatments. SANAD II therefore provides much needed longer-term head-to-head data to better inform treatment policy and guidance.

Results from SANAD II have important implications for clinical practice and research. Although levetiracetam and zonisamide are licensed for used as monotherapy in patients with focal epilepsy in Europe and worldwide, our results do not support their use as first-line monotherapy. This finding is most relevant to levetiracetam, which has become a commonly prescribed first-line anti-seizure medication because of its ease of titration and assumed efficacy and tolerability. Further studies are now required to assess the clinical effectiveness and cost-effectiveness of other anti-seizure medications (eg, lacosamide, brivaracetam, and perampanel) and the design of future studies should be debated given that the SANAD I and SANAD II trials provide historical control data.

## Data sharing

All requests for data sharing should be addressed to the trial co-sponsors. Co-sponsors will process the requests by involving all applicable parties in their decision making outcome (eg, joint data controllers and pharmaceutical companies). Data sharing packs are prepared at the end of trial and involve as a minimum all versions of the trial protocol, all versions of the annotated trial case report forms, and dataset.

## Declaration of interests

AM reports grants from the National Institute for Health Research Health Technology Assessment, during the conduct of the study, as well as grants from UCB Pharma, outside of the submitted work. JPL reports grants from University of Liverpool during the conduct of the study; grants and personal fees from UCB Pharma; and personal fees from Eisai, Janssen CIlag Pharmaceuticals, GW Pharmaceuticals, GSK Pharma, outside of the submitted work. GS reports personal fees from UCB Pharma, Eisai, Arvelle Therapeutics GmbH, outside of the submitted work. CP reports grants from National Institute for Health and Care Research Health Technology Assessment Programme during the conduct of this study. CT reports grants from University of Liverpool during the conduct of the study. DH reports grants from National Institute for Health Research Health Technology Assessment Programme during the conduct of the study. RM reports personal fees from UCB Pharma and grants from UCB Pharma and Sanofi, outside of the submitted work. PES is a member of the NICE Panel for Epilepsy guideline 2021 and is an editor of the journal Practical Neurology. All other authors declare no competing interests.
