# Department of Error

**DOI:** 10.1016/S0140-6736(21)01012-6

**Published:** 2021-05-15

*Marson A, Burnside G, Appleton R, et al. The SANAD II study of the effectiveness and cost-effectiveness of levetiracetam, zonisamide, or lamotrigine for newly diagnosed focal epilepsy: an open-label, non-inferiority, multicentre, phase 4, randomised controlled trial.* Lancet *2021; **397:** 1363–74*—In this Article, N Vora should not have been included in the SANAD II collaborators list. This correction has been made to the online version as of May 13, 2021.

