# Supplementary material for: The SANAD II study of the effectiveness and cost-effectiveness of levetiracetam, zonisamide, or lamotrigine for newly diagnosed focal epilepsy: an open-label, non-inferiority, multicentre, phase 4, randomised controlled trial
Source: Lancet. 2021 Apr 10;397(10282):1363–74. doi: 10.1016/S0140-6736(21)00247-6 (PMC8047799; doi:10.1016/S0140-6736(21)00247-6)
Supplement: Supplementary appendix 2 [file mmc2.pdf]

THE LANCET

Supplementary appendix

This appendix formed part of the original submission and has been peer reviewed. We post it as supplied by the authors.

Supplement to: Marson A, Burnside G, Appleton R, et al. The SANAD II study of the effectiveness and cost-effectiveness of levetiracetam, zonisamide, or lamotrigine for newly diagnosed focal epilepsy: an open-label, non-inferiority, multicentre, phase 4, randomised controlled trial. *Lancet* 2021; **397:** 1363–74.

Health Economics Analysis Plan (HEAP) for the SANAD-II trial

A pragmatic randomised controlled trial comparing the effectiveness and cost effectiveness of levetiracetam and zonisamide versus standard treatments for epilepsy: a comparison of Standard And New Antiepileptic Drugs (SANAD-II)

HEAP Version: Final 1.0 Dated: 03/05/2019

Trial information

EudraCT number: 2012-001884-64

ISRCTN number: 30294119

Funder reference: NIHR HTA – 09/144/09

Sponsor reference: UoL000314

Study Sponsors

The Walton Centre NHS Foundation Trust
Lower Lane
Fazakerley
Liverpool
L9 7LJ

University of Liverpool
Research Business Services
Foresight Building
3 Brownlow Street
Liverpool
L69 3GL

Date of approval: DD/MM/YYYY

Date of approval: DD/MM/YYYY

The HEAP was prepared by Dr Catrin Plumpton (HE researcher) and approved by Prof Dyfrig Hughes (lead HE) and Prof Tony Marson (Chief Investigator). The trial health economists are responsible for conducting and reporting the economic evaluation in accordance with the HEAP. Dr Catrin Plumpton will conduct, and Prof Dyfrig Hughes will oversee the health economic analysis.

The purpose of the HEAP is to describe the analysis and reporting procedure intended for the economic analyses to be undertaken. All items noted apply to both Arm A and Arm B, analysed separately, unless explicitly noted otherwise. The HEAP is designed to ensure that there is no conflict with the protocol and associated SAP and it should be read in conjunction with them.

Protocol version: 8 Dated 28/11/2018

SAP version: 1.0 Dated 28/01/2019

Signatures

Dr Catrin Plumpton (HE researcher) Date: 8th May 2019

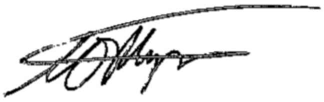

Prof Dyfrig Hughes (lead HE) Date: 8th May 2019

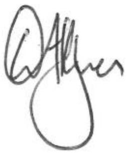

Prof Tony Marson (Chief Investigator) Date: 9th May 2019

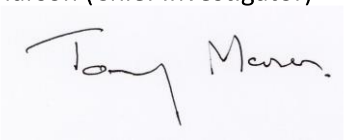

HEAP revisions

Protocol version	Updated HEAP version	Section number changed	Description of and reason for change	Individual making the change	Date changed

Contents

Glossary 5

1. Economic Approach/Overview 6
   - 1.1. Aims and objectives of economic evaluation 6
   - 1.2. Overview of economic analysis 6
   - 1.3. Jurisdiction and Perspective 6
   - 1.4. Time horizon 6
2. Economic Data Collection and Management 6
   - 2.1. Monitoring collection of health economic data 6
   - 2.2. Database management 6
   - 2.3. Data entry 6
   - 2.4. Data validation and cleaning 6
   - 2.5. Data archiving 7
   - 2.6. Statistical software used for HE analysis 7
   - 2.7. Identification of resources 7
   - 2.8. Measurement of resource use data 7
   - 2.9. Valuation of resource use data 8
   - 2.10. Identification of outcome(s) 8
   - 2.11. Measurement of outcomes 8
   - 2.12. Valuation of outcomes 9
3. Economic Data Analysis 9
   - 3.1. Analysis population 9
   - 3.2. Timing of analysis 10
   - 3.3. Discount rates for costs and benefits 10
   - 3.4. Cost-effectiveness thresholds 10
   - 3.5. Statistical decision rules 10
   - 3.6. Analysis of resource use 10
   - 3.7. Analysis of costs 10
   - 3.8. Analysis of outcomes 10
   - 3.9. Missing data 11
   - 3.10. Analysis of cost effectiveness 11
   - 3.11. Summary of base case analysis 12
   - 3.12. Sampling uncertainty 12
   - 3.13. Subgroup analyses/Analysis of heterogeneity 12
   - 3.14. Sensitivity analyses 12
   - 3.15. Post hoc analyses 12

4. Reporting/Publishing 12
4.1. Responsibility for health economic results and reporting 12
4.2. Reporting standards 12
4.3. Reporting deviations from the HEAP 12
5. Document location 13
6. References 13
7. Appendices 15
7.1. Appendix 1: CHEERS checklist 15
7.2. Appendix 2: Resource use questionnaire 18

Glossary

AED	Anti-epileptic Drugs
AUC	Area Under the Curve
BNF	British National Formulary
CEAC	Cost Effectiveness Acceptability Curve
CHEERS	Consolidated Health Economic Evaluation Reporting Standards
CRF	Case Report Form
CSRI	Client Service Receipt Inventory
GDP	Gross Domestic Product
HE	Health Economist
HES	Hospital Episode Statistics
HRG	Health Resource Group
ICER	Incremental Cost Effectiveness Plane
ITT	Intention To Treat
ONS	Office for National Statistics
NHS	National Health Service
NICE	National Institute for health and Care Excellence
PCA	Prescription Cost Analysis
PSS	Personal Social Services
QALY	Quality-Adjusted Life Year
RCT	Randomised Controlled Trial
SAIL	Secure Anonymised Information Linkage

1. Economic Approach/Overview

1.1. Aims and objectives of economic evaluation

Arm A: To estimate the long-term cost-effectiveness of lamotrigine, levetiracetam and zonisamide in patients with newly treated focal onset seizures.

Arm B: To estimate the long-term cost-effectiveness of levetiracetam and valproate in patients with newly treated generalised onset seizures or seizures that are difficult to classify.

1.2. Overview of economic analysis

The within-trial economic analysis will be performed using individual patient-level data from the SANAD-II trial. The analytical approach will take the form of cost-utility analysis. Based on trial evidence, incremental cost effectiveness ratios (expressed as cost per Quality-Adjusted Life Year (QALY) gained) will be calculated by taking a ratio of the difference between treatments in the mean costs and mean QALYs. A cost-effectiveness analysis will also be undertaken, based on the outcome of 12-month remission.

1.3. Jurisdiction and Perspective

The trial is conducted in the UK which has National Health Services (NHS), providing publicly funded healthcare, primarily free of charge at the point of use. The economic analysis will be from the NHS and personal social services (PSS) perspective.

1.4. Time horizon

The primary economic analysis will compare the costs and consequences of each treatment within each arm over the first 24 months after randomisation. A secondary analysis will be based on the extended follow-up to compare costs and benefits over a 48-month follow-up period from randomisation.

2. Economic Data Collection and Management

2.1. Monitoring collection of health economic data

Trial health economists will work closely with the trial team throughout the data collection period. Data collection forms will be assessed throughout the trial period to monitor the quality of the data and amend any forms or procedures, if necessary.

2.2. Database management

Economic data will be stored securely on the trial database and managed by the trial database manager.

2.3. Data entry

All data will be entered by the central research team. Baseline questionnaires will be forwarded from the recruitment site to the central research team; follow-up data collected by postal questionnaires will be returned to the central research team. The database will use controls to limit data entry to plausible values.

2.4. Data validation and cleaning

Face validity tests will be conducted on data (e.g. to identify numerical outliers or misspelt text) and checked against the source documents. Corrections identified will be documented.

2.5. Data archiving

A copy of HE analysis files, derived datasets, interim datasets and final analysis will be locked and archived. Archived datasets will be held in Bangor University and will conform to the University's data security policy and data compliance and GDPR policies.

2.6. Statistical software used for HE analysis

Statistical analyses will be carried out in StataIC version 13 or later (StataCorp LLc, College Station, TX).

2.7. Identification of resources

The use of resources in primary, secondary and community care services, and medication costs relating to trial and concomitant medications will be measured. Indirect costs (relating to income and hours worked) were collected based on earlier versions of the protocol (this changed in Substantial Amendment 7).

2.8. Measurement of resource use data

The measurement of resource use is based on complementary approaches using data collected as part of the trial and as part of routine care. Participants' use of hospital services will be obtained from:

For participants aged ≥16

- Postal questionnaires including a modified Client Service Receipt Inventory (CSRI) based on that from the SANAD trial [1, 2] completed by the participant (administered at 3 months, 6 months, 12 months, and annually thereafter) to collect information on health service resource use and medicines use.
- Protocol v7 onwards (Substantial Amendment 16): Questionnaires will be provided during outpatient visits.

For participants aged <16

- Postal questionnaires including a modified CSRI completed by the parent/carer (administered at 3 months, 6 months, 12 months, and annually thereafter) to collect information on health service resource use and medicines use.
- Protocol v7 onwards: Questionnaires will be provided during outpatient visits.

For all participants

- Hospital Episode Statistics (HES) data relating to participants' use of secondary care will be requested from NHS Digital [3] (for patients in England) and the Secure Anonymised Information Linkage (SAIL) databank [4] (for patients in Wales) by the central research team. HES data contain details of all admissions to NHS hospitals in England and Wales, and provide HRGs on the type of care patients receive at a ward-level, outpatient visits and A&E admissions, but do not provide details on locally-reimbursed costs such as CT scans, ICU and HDU stays. Earlier protocol versions (versions 3 and 4) mentioned patient administration systems (e.g. Patient Level Information and Costing System) data, but this approach was impractical due to the number of hospitals involved.
- Where neither HES data nor self-report data are available, resources triggered by adverse events will be captured in the follow-up CRF for each patient experiencing an adverse event requiring hospitalisation.

All resource use will be measured irrespective of whether they are related to epilepsy.

2.9. Valuation of resource use data

All resource use will be valued in monetary terms using appropriate UK unit costs or participant valuations estimated at the time of analysis (cost year: 2018/19). Adjustments will be made for inflation if necessary using the Hospital Price Index according to the current version of the compendium of Unit Costs of Health and Social Care [5].

HRGs will be used as the main currency of the economic analysis for inpatient stays with cost codes allocated based on the latest available National Schedule [6] and National Tariff [7]. Obsolete National Tariff and Schedule codes will be uplifted using the Hospital Price Index [5]. This resource will also be the source of unit costs which will be applied to primary health care and outpatient contacts.

Bundled National Tariff costs will be based on the hospital spell and incorporated excess ward days and whether the case was elective or emergency. Tariff codes will be obtained primarily from HES data but if unavailable, they will be assigned by reference to CRFs and an appropriate HRG code will be assigned. Similarly, appropriate HRGs will be applied to unassignable National Tariff HRG codes (such as UZ01C and WA14Z) appearing in the HES data.

Unbundled costs will be assigned using information recorded in CRFs (e.g. adverse events CRF). Appropriate HRGs will be assigned and the cost calculated from the National Schedule codes [6]. In the absence of any higher cost code indicators, a basic code will be applied from the National Schedule of Reference Costs.

Medication costs will be taken from the British National Formulary (BNF) [8] and the Prescription Costs Analysis (PCA) for England [9].

2.10. Identification of outcome(s)

The primary economic outcome measure will be Quality-Adjusted Life Years (QALYs), generated from utility data measured using the EQ-5D-3L [10]. Secondary economic outcome measures will be the EQ-VAS [10], an epilepsy-specific utility measure based on the NEWQOL-6D questionnaire [11], and effectiveness outcomes aligned to the clinical analysis.

2.11. Measurement of patient-reported outcomes

For participants aged ≥16

- Baseline questionnaire will be completed by the participant during the baseline visit.
- Postal questionnaires will be completed by the participant 12 months and annually thereafter.
- Protocol v7 onwards: Questionnaires will be provided during outpatient visits.

For participants aged 8-15

- Baseline questionnaire will be completed by the participant during the baseline visit.
- Baseline questionnaires will be completed by proxy (parent / carer) during the baseline visit.
- Postal questionnaires will be completed by the participant 12 months and annually thereafter.
- Postal questionnaires will be completed by proxy (parent / carer) 12 months and annually thereafter.

- Protocol v7 onwards: Questionnaires will be provided during outpatient visits.

For participants aged 5-7

- Baseline questionnaires will be completed by proxy (parent / carer) during the baseline visit.
- Postal questionnaires will be completed by proxy (parent / carer) 12 months and annually thereafter.
- Protocol v7 onwards: Questionnaires will be provided during outpatient visits.

2.12. Valuation of outcomes

For participants aged ≥16

- Utility scores will be obtained using the EQ-5D-3L questionnaire and applying UK tariffs.
- Overall health will be derived from the EQ-VAS
- Epilepsy-specific utility scores will be obtained from the NEWQOL-6D using UK tariffs [13].

For participants aged 8-15

- Utility scores will be obtained using the EQ-5D-3L-Y questionnaire and applying UK (adult) tariffs.
- Utility scores will be obtained using the EQ-5D-3L questionnaire completed by proxy (parent or carer) using UK (adult) tariffs.
- Overall health will be derived from the EQ-VAS
- Epilepsy-specific utility scores will be obtained from the NEWQOL-6D completed by proxy (patient or carer) using UK tariffs.

For participants aged 5-7

- Utility scores will be obtained using the EQ-5D-3L questionnaire completed by proxy (parent or carer) using UK (adult) tariffs.
- Overall health will be derived from the EQ-VAS
- Epilepsy-specific utility scores will be obtained from the NEWQOL-6D completed by proxy (patient or carer) using UK tariffs.

For all participants

- Clinical effectiveness outcomes will be obtained from CRF data

3. Economic Data Analysis

3.1. Analysis population

Arms A and B will be analysed separately. The primary analyses will include all participants (children and adults).

Full analysis set: All randomised participants, which is in accordance with the "intention to treat" (ITT) principle.

3.2. Timing of analysis

Analysis for each arm will be conducted once data for that arm is locked. A single HES data application will be made to obtain data for all participants, requesting data until the end of follow-up, or the end of the 2018/19 financial year, whichever is first.

3.3. Discount rates for costs and benefits

Costs and benefits (after the 1st year) will be discounted at the NICE recommended rate of 3.5% per annum [12].

3.4. Cost-effectiveness thresholds

The estimated mean QALYs and costs associated with each treatment option will be combined with a feasible range of values for decision makers' cost-effectiveness threshold (ʎ), to obtain distribution of net benefits at different levels of ʎ. The primary economic analysis will use the NICE specified cost-effectiveness threshold of £20,000 per QALY.

3.5. Statistical decision rules

Due to multiplicity, for Arm A, 97.5% two-sided CIs will be reported. For Arm B, 95% CIs will be reported.

3.6. Analysis of resource use

HES data will be the primary source of secondary care resource use, supplemented by self-report data. Resource use in primary care and community care services will be based on self-report. Resource use, with bootstrapped central range, will be tabulated by trial arm in order to describe differences in the use of services between randomised groups.

3.7. Analysis of costs

Within-trial total costs for each patient will be calculated from the sum of all costs (associated with primary, secondary and community care services, and medication use).

Costs at baseline, relating to the 3-months preceding randomisation, will be calculated from HES data in order to adjust for any baseline difference [14]. This will only relate to all secondary care usage. If a hospitalisation is observed for the period subsequent to randomisation, an adjustment may be necessary to apportion costs given that ward costs relate to episodes of care which could start prior to randomisation.

Total costs during the course of the trial will be calculated for the intention to treat population, with summary statistics generated by intervention group. Differences between intervention groups will be compared with reference to bootstrapped central range, based on 10,000 replicates.

In order to account for any imbalances in important clinical or demographic variables, we will implement regression analysis. The suitability of OLS is likely given the large sample size, however this will be determined once the data has been received; in the event that OLS is not deemed suitable, generalised linear models will be used as an alternative [15].

3.8. Analysis of outcomes

For participants aged ≥16

- Based on utilities derived from the EQ-5D-3L, a QALY profile over the trial period will be estimated based on the area under the curve (AUC), assuming the trapezoidal rule.
- Based on responses to the EQ-VAS, a QALY profile over the trial period will be estimated based using the AUC approach.

- Utility scores derived from the NEWQOL-6D will also be analysed using an AUC approach.

For participants aged 8-15

- Based on utilities derived from the EQ-5D-3L (self-report, supplemented where necessary by proxy responses), a QALY profile over the trial period will be estimated based on the AUC, assuming the trapezoidal rule.
- Based on responses to the EQ-VAS, a QALY profile over the trial period will be estimated based using the AUC approach.
- Utility scores derived from the NEWQOL-6D (proxy responses) will also be analysed using an AUC approach.

For participants aged 5-7

- Based on utilities derived from the EQ-5D-3L (proxy responses), a QALY profile over the trial period will be estimated based on the AUC, assuming the trapezoidal rule.
- Based on responses to the EQ-VAS, a QALY profile over the trial period will be estimated based using the AUC approach.
- Utility scores derived from the NEWQOL-6D (proxy responses) will also be analysed using an AUC approach.

An appropriate regression model will be used to adjust for any imbalance in baseline utility (however small), baseline characteristics, and the minimisation variables of the randomisation process, if required, dummy variables will be used to indicate whether EQ-5D-3L data was collected via self-report or proxy measure [16].

For all participants

Clinical effectiveness outcomes will be taken from the clinical analysis.

3.9. Missing data

Trial data will be examined for any missing data. The appropriate method for dealing with missing data will depend on the share of missing data and likely mechanism of missingness. For example multiple imputation methods may be used if the data is missing at random (MAR). In the event that data is not MAR, appropriate alternatives will be considered [17].

3.10. Analysis of cost effectiveness

Cost and QALY data will be combined to calculate incremental cost-effectiveness ratios (ICERs).

For both arms:

Antiepileptic drugs will be ranked according to their effectiveness (QALYs). Antiepileptic drugs which are dominated (higher cost and lower QALY) will be removed.

ICERs will be calculated as

$$ICER = \Delta Costs / \Delta QALY$$

Where for the first calculation, ΔCosts is the difference in total costs between AED_1 and AED_2; and ΔQALY is the difference in QALYs between AED_1 and AED_2

Dominance and extended dominance will be reported, following standard definitions.

Cost effectiveness for clinical outcomes will be calculated using

$$ICER = \Delta Costs / \Delta Outcome$$

3.11. Summary of base case analysis

The base case analysis will be conducted from the perspective of the NHS and personal social services, over a time horizon of two years. QALYs will be based on the EQ-5D patient reported scores where available, and proxy-reported for children aged 7 and under. The base case will be defined using the imputed data set, with costs and QALYs adjusted for covariates.

3.12. Sampling uncertainty

The joint uncertainty in incremental costs and QALYs will be represented as a cost-effectiveness plane, and as a cost-effectiveness acceptability curve (CEAC), illustrating the probability of each AEDs being cost effective for given cost-effectiveness thresholds [18].

3.13. Subgroup analyses/Analysis of heterogeneity

Subgroup analysis will be conducted on the final data sets to investigate how cost-effectiveness varies by subgroup. Subgroups considered will include children (age <16) and adults (age ≥16).

Heterogeneity will be analysed using appropriate regression based methods.

3.14. Sensitivity analyses

Sensitivity analyses will be undertaken to explore key uncertainties in the economic evaluation. The results for available case cost and QALY data (i.e. those with no missing data) will be provided to identify the impact of missing data.

A further sensitivity analysis will replicate the base case analysis, using the AUC derived from the EQ-VAS and the NEWQOL-6D outcome measure.

Cost-effectiveness analyses will be conducted to align with the clinical outcomes.

A sensitivity cost-effectiveness analysis will be conducted using an extended time horizon for participants who completed 48-months follow-up post randomisation.

3.15. Post hoc analyses

We will identify and clearly record any post hoc analyses.

4. Reporting/Publishing

4.1. Responsibility for health economic results and reporting

The following HEs [Prof Dyfrig Hughes, Dr Catrin Plumpton] have overall responsibility for the production and reporting of the results of the economic evaluation. The HEs are responsible for checking that the results for any outcomes reported in the economic evaluation are consistent and accurate. Any differences in results are to be raised with the Trial Statistician before being reported.

4.2. Reporting standards

The CHEERS guidelines will be followed when reporting the health economic evaluation [19].

4.3. Reporting deviations from the HEAP

Any deviation from HEAP will be described and justified in the final report (HTA monograph).

5. Document location

The master file is held at CTRC, University of Liverpool. The statistical master file holding details of the randomisation process and relevant protocol deviations is held at CTRC, University of Liverpool

6. References

1. Beecham J, Knapp M. Costing psychiatric interventions. In: G Thornicroft, editor. Measuring Mental Health Needs. 2nd ed. London: Gaskell; 2001. p. 200-24
2. Marson AG, Appleton R, Baker, GA et al. A randomised controlled trial examining the longer-term outcomes of standard versus new antiepileptic drugs. The SANAD trial. Health Technology Assessment 2007; 11(37).
3. NHS Digital Data Linkage & Extract Service. Available from: https://digital.nhs.uk/
4. The Secure Anonymised Information Linkage databank. Available from: https://saildatabank.com/
5. Curtis L, Burns A. Unit costs of health and social care 2018. University of Kent at Canterbury: Personal Social Services Research Unit. Available at: https://www.pssru.ac.uk/project-pages/unit-costs/unit-costs-2018/
6. Department of Health. NHS reference costs 2017-18. Available online at: https://improvement.nhs.uk/resources/reference-costs/
7. National Tariff 2017-2018. Available from: https://improvement.nhs.uk/resources/national-tariff/
8. Joint Formulary Committee. British National Formulary available from https://bnf.nice.org.uk/
9. NHS Digital, "Prescription Cost Analysis England 2018,". [Online]. Available: https://digital.nhs.uk/data-and-information/publications/statistical/prescription-cost-analysis/2018
10. Kind P. The EuroQoL instrument: an index of health-related quality of life. In: Spilker, B, Quality of Life and Pharmacoeconomics in Clinical Trials, Philadelphia: Lippincott-Raven, 1996.
11. Jacoby A, Baker G, Smith D, Dewey M, Chadwick D. Measuring the impact of epilepsy: the development of a novel scale. Epilepsy Res. [Research Support, Non-U.S. Gov't]. 1993,Sep;16(1):83-8
12. National Institute for Health and Care Excellence. Guide to the methods of technology appraisal. Process and methods [PMG9]. 2013.
13. Dolan P. Modeling valuations for EuroQol health states. Med Care 1997; 35(11): 1095-108.
14. van Asselt AD, van Mastrigt GA, Dirksen CD, Arntz A, Severens JL, Kessels AG. How to deal with cost differences at baseline. Pharmacoeconomics 2009; 27 (6): 519-528
15. Mihaylova B, Briggs A, O'Hagan A, Thompson SG. Review of statistical methods for analysing healthcare resources and costs. Health Econ. 2011 Aug;20(8):897-916.
16. Manca A, Hawkins N, Sculpher MJ. Estimating mean QALYs in trial-based cost effectiveness analysis: the importance of controlling for baseline utility. Health Econ. 2005;14(5):487-496.
17. Leurent B, Gomes M, Faria R, Morris S, Grieve R, Carpenter JR. Sensitivity analysis for Not-at-Random missing data in trial-based cost-effectiveness analysis: A tutorial. Pharmacoeconomics 2018
18. Fenwick E, Claxton K, Sculpher M. Representing uncertainty: the role of cost-effectiveness acceptability curves. Health Econ. 2001 Dec;10(8):779-87.

19. Husereau D, Drummond M, Petrou S, Carswell C, Moher D, Greenberg D, Augustovski F, Briggs AH, Mauskopf J, Loder E. Consolidated health economic evaluation reporting standards (CHEERS) statement. Cost Effectiveness and Resource Allocation. 2013 Mar 25;11(1):6.

7. Appendices

7.1. Appendix 1: CHEERS checklist

Section/item	Item No	Recommendation
**Title and abstract**		
Title	1	Identify the study as an economic evaluation or use more specific terms such as “cost-effectiveness analysis”, and describe the interventions compared.
Abstract	2	Provide a structured summary of objectives, perspective, setting, methods (including study design and inputs), results (including base case and uncertainty analyses), and conclusions.
**Introduction**		
Background and objectives	3	Provide an explicit statement of the broader context for the study.<br>Present the study question and its relevance for health policy or practice decisions.
**Methods**		
Target population and subgroups	4	Describe characteristics of the base case population and subgroups analysed, including why they were chosen.
Setting and location	5	State relevant aspects of the system(s) in which the decision(s) need(s) to be made.
Study perspective	6	Describe the perspective of the study and relate this to the costs being evaluated.
Comparators	7	Describe the interventions or strategies being compared and state why they were chosen.
Time horizon	8	State the time horizon(s) over which costs and consequences are being evaluated and say why appropriate.
Discount rate	9	Report the choice of discount rate(s) used for costs and outcomes and say why appropriate.
Choice of health outcomes	10	Describe what outcomes were used as the measure(s) of benefit in the evaluation and their relevance for the type of analysis performed.
Measurement of effectiveness	11a	*Single study-based estimates:* Describe fully the design features of the single effectiveness study and why the single study was a sufficient source of clinical effectiveness data.
	11b	*Synthesis-based estimates:* Describe fully the methods used for identification of included studies and synthesis of clinical effectiveness data.
Measurement and valuation of preference based outcomes	12	If applicable, describe the population and methods used to elicit preferences for outcomes.

Estimating resources and costs	13a	*Single study-based economic evaluation:* Describe approaches used to estimate resource use associated with the alternative interventions. Describe primary or secondary research methods for valuing each resource item in terms of its unit cost. Describe any adjustments made to approximate to opportunity costs.
	13b	*Model-based economic evaluation:* Describe approaches and data sources used to estimate resource use associated with model health states. Describe primary or secondary research methods for valuing each resource item in terms of its unit cost. Describe any adjustments made to approximate to opportunity costs.
Currency, price date, and conversion	14	Report the dates of the estimated resource quantities and unit costs. Describe methods for adjusting estimated unit costs to the year of reported costs if necessary. Describe methods for converting costs into a common currency base and the exchange rate.
Choice of model	15	Describe and give reasons for the specific type of decision-analytical model used. Providing a figure to show model structure is strongly recommended.
Assumptions	16	Describe all structural or other assumptions underpinning the decision-analytical model.
Analytical methods	17	Describe all analytical methods supporting the evaluation. This could include methods for dealing with skewed, missing, or censored data; extrapolation methods; methods for pooling data; approaches to validate or make adjustments (such as half cycle corrections) to a model; and methods for handling population heterogeneity and uncertainty.
**Results**		
Study parameters	18	Report the values, ranges, references, and, if used, probability distributions for all parameters. Report reasons or sources for distributions used to represent uncertainty where appropriate. Providing a table to show the input values is strongly recommended.
Incremental costs and outcomes	19	For each intervention, report mean values for the main categories of estimated costs and outcomes of interest, as well as mean differences between the comparator groups. If applicable, report incremental cost-effectiveness ratios.
Characterising uncertainty	20a	*Single study-based economic evaluation:* Describe the effects of sampling uncertainty for the estimated incremental cost and incremental effectiveness parameters, together with the impact of methodological assumptions (such as discount rate, study perspective).
	20b	*Model-based economic evaluation:* Describe the effects on the results of uncertainty for all input parameters, and uncertainty related to the structure of the model and assumptions.

Characterising heterogeneity	21	If applicable, report differences in costs, outcomes, or cost-effectiveness that can be explained by variations between subgroups of patients with different baseline characteristics or other observed variability in effects that are not reducible by more information.
**Discussion**		
Study findings, limitations, generalisability, and current knowledge	22	Summarise key study findings and describe how they support the conclusions reached. Discuss limitations and the generalisability of the findings and how the findings fit with current knowledge.
**Other**		
Source of funding	23	Describe how the study was funded and the role of the funder in the identification, design, conduct, and reporting of the analysis. Describe other non-monetary sources of support.
Conflicts of interest	24	Describe any potential for conflict of interest of study contributors in accordance with journal policy. In the absence of a journal policy, we recommend authors comply with International Committee of Medical Journal Editors recommendations.

7.2. Appendix 2: Resource use questionnaire

Deposited in DIRUM (http://www.dirum.org/instruments/details/93
