# Supplementary material for: The SANAD II study of the effectiveness and cost-effectiveness of levetiracetam, zonisamide, or lamotrigine for newly diagnosed focal epilepsy: an open-label, non-inferiority, multicentre, phase 4, randomised controlled trial
Source: Lancet. 2021 Apr 10;397(10282):1363–74. doi: 10.1016/S0140-6736(21)00247-6 (PMC8047799; doi:10.1016/S0140-6736(21)00247-6)
Supplement: Supplementary appendix 3 [file mmc3.pdf]

THE LANCET

Supplementary appendix

This appendix formed part of the original submission and has been peer reviewed. We post it as supplied by the authors.

Supplement to: Marson A, Burnside G, Appleton R, et al. The SANAD II study of the effectiveness and cost-effectiveness of levetiracetam, zonisamide, or lamotrigine for newly diagnosed focal epilepsy: an open-label, non-inferiority, multicentre, phase 4, randomised controlled trial. *Lancet* 2021; **397:** 1363–74.

Web appendix

The SANAD II study of the effectiveness and cost-effectiveness of levetiracetam, zonisamide, or lamotrigine for newly diagnosed focal epilepsy: an open-label, non-inferiority, multicentre, phase 4, randomised controlled trial

Table 1. HR estimates for time to 12-month remission

**Model and analysis set**	**Lamotrigine versus levetiracetam HR (97·5% CI)**	**Lamotrigine versus zonisamide HR (97·5% CI)**
Primary analysis: Cox model with treatment (ITT)	1·18 (0·96 to 1·47)	1·03 (0·83 to 1·28)
Cox model with treatment (ITT), gender, number of seizures and centre as random effect	1·13 (0·91 to 1·41)	1·01 (0·81 to 1·25)
Fine and Gray model with treatment (PP)	1·32[1] (1·05 to 1·66)	1·37[1] (1·08 to 1·73)

HR> 1 indicates benefit to lamotrigine. ITT: Intention to treat. PP: Per Protocol

[1] Ratio of rate of occurrence of 12-month remission in patients who are currently event free or who have previously failed randomised treatment

Table 2. Annual time to 12-month remission probability estimates from Kaplan Meier analysis

	Events/ total	Year 1	Year 2	Year 3	Year 4	Year 5
Number at risk						
Lamotrigine	222/330	291	92	34	12	2
Levetiracetam	204/332	293	107	57	22	5
Zonisamide	209/328	284	92	29	10	2
Percentage 12-month remission (95% CI)						
Lamotrigine		34 (29,39)	63 (58, 69)	79 (74, 84)	82 (77, 88)	86 (80, 92)
Percentage 12-month remission (95% CI)						
Levetiracetam		37 (32, 43)	59 (53, 64)	70 (64, 76)	77 (71, 82)	79 (73, 85)
Percentage 12-month remission (95% CI)						
Zonisamide		35 (29, 40)	63 (57, 68)	78 (72, 84)	84 (78, 90)	91 (83, 100)
Difference in percentage of 12-month remission compared with Lamotrigine (95% CI)						
Levetiracetam		3 (-5, 11)	-5 (-13, 3)	-9 (-17, -2)	-6 (-14, 2)	-7 (-16, 1)
Zonisamide		1 (-7, 9)	-1 (-9, 7)	-1 (-9, 7)	2 (-6, 10)	5 (-5, 16)

Web figure 1. Cumulative incidence of time to 12 month remission from per protocol analysis: Lamotrigine versus levetiracetam versus zonisamide.

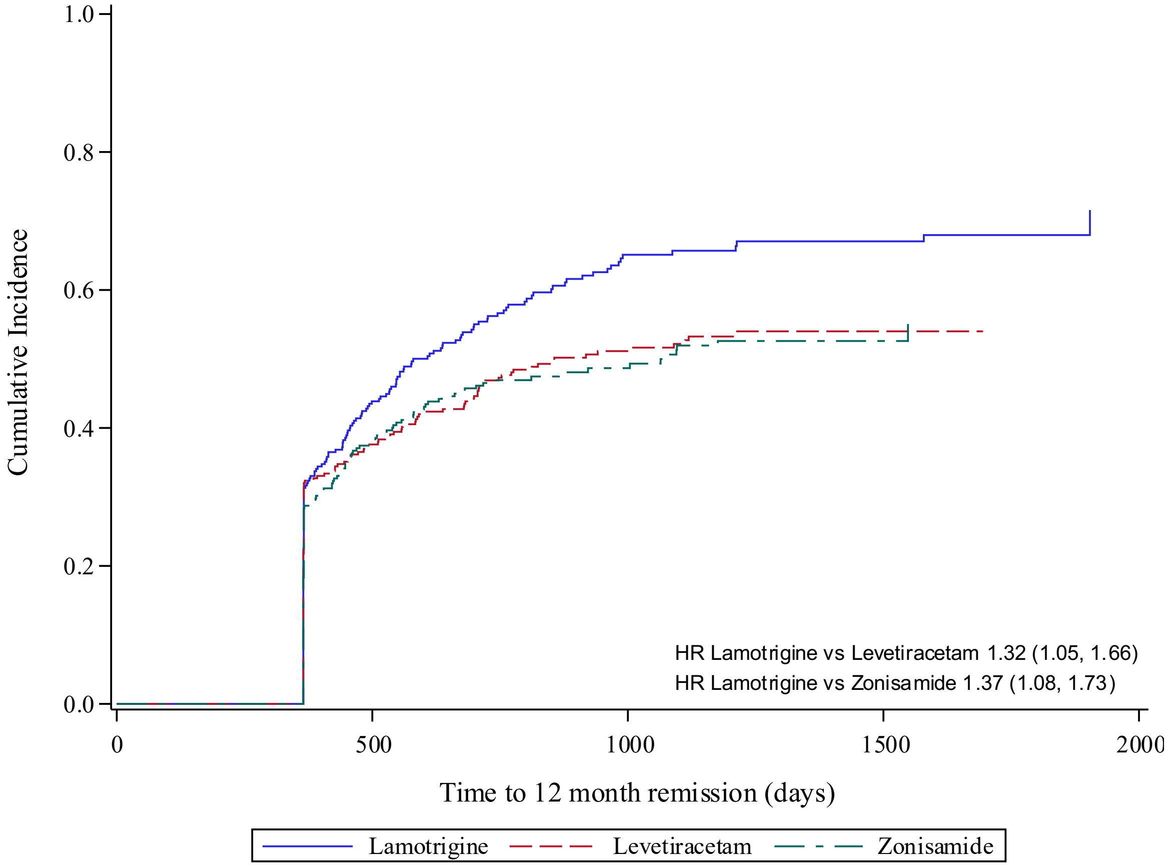

Web figure 2. Kaplan Meier plot of time to 24 month remission: Lamotrigine versus levetiracetam versus zonisamide.

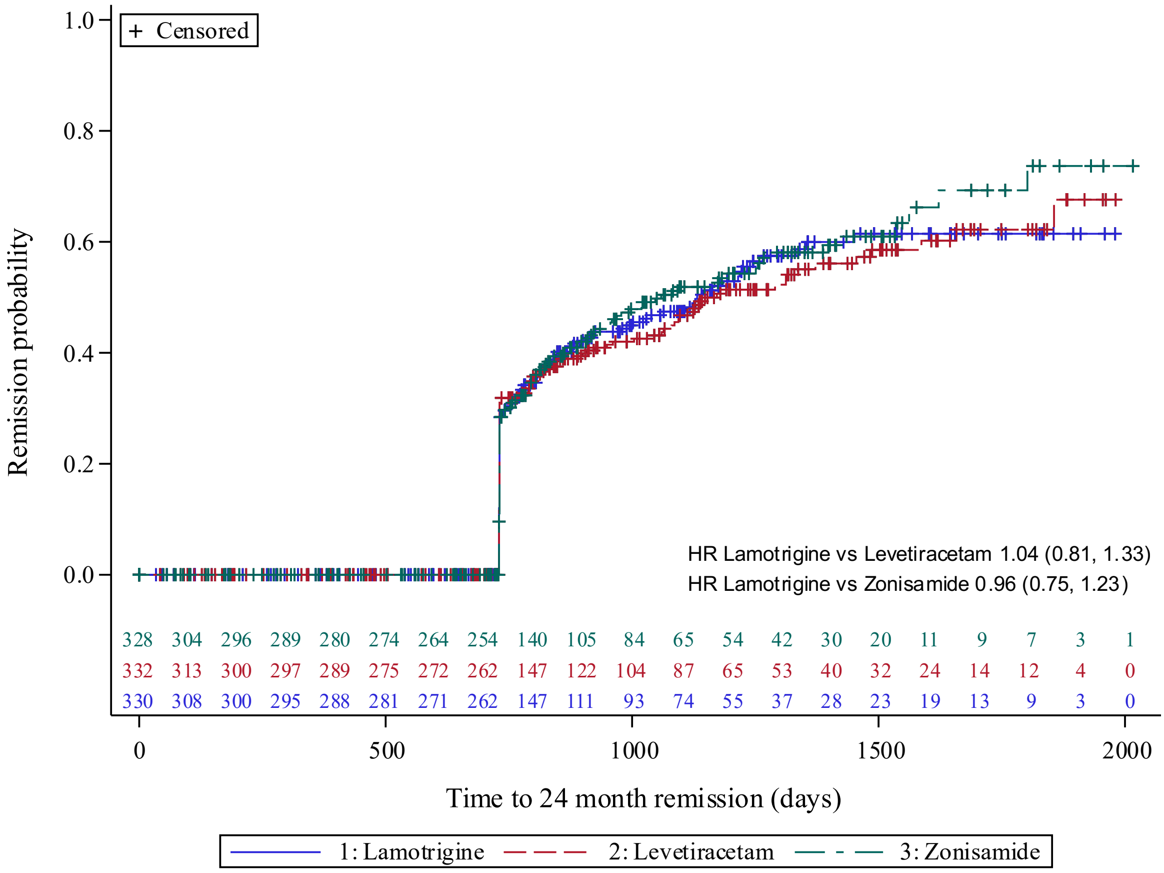

Web figure 3. Kaplan Meier plot of time to first seizure: Lamotrigine versus levetiracetam versus zonisamide.

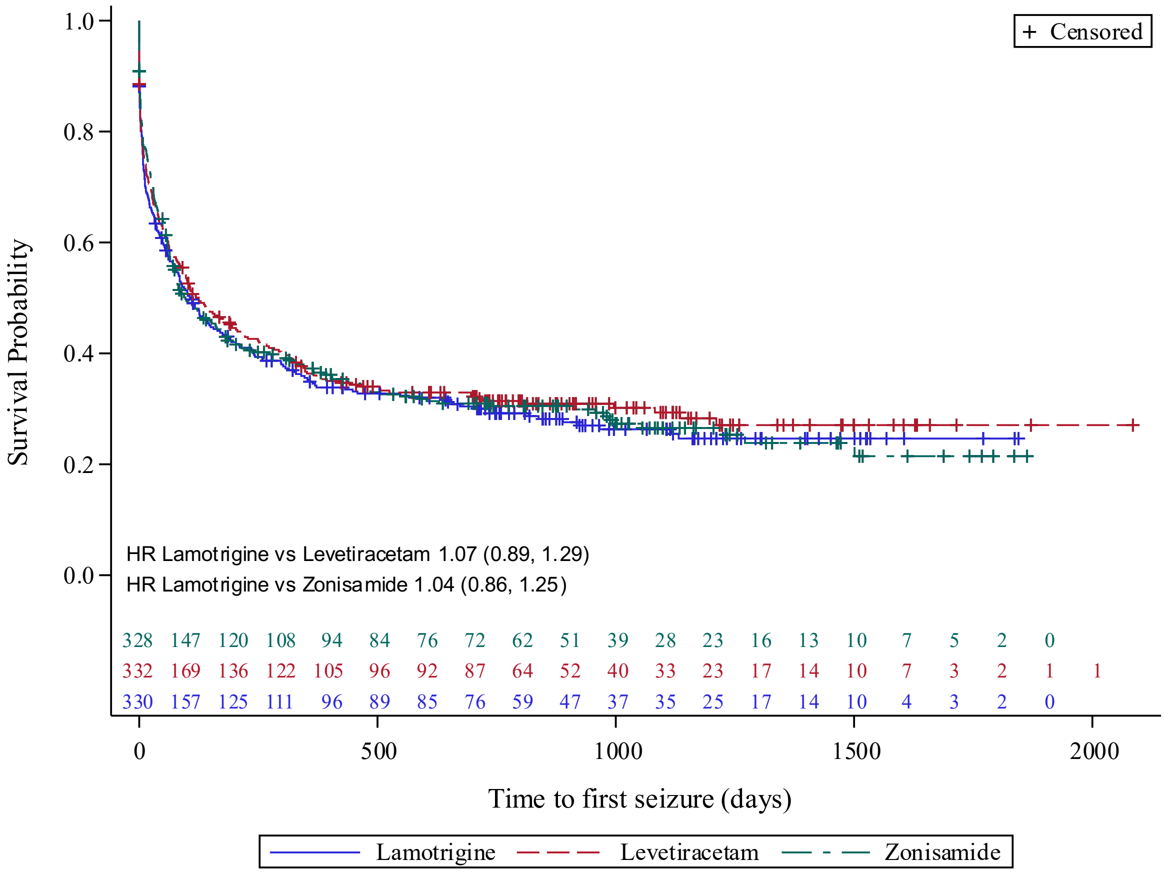

Table 3. Time to treatment failure probabilities from Kaplan Meier analysis

	Events/ total	Year 1	Year 2	Year 3	Year 4	Year 5
Number at risk						
Lamotrigine	97/330	241	192	101	36	8
Levetiracetam	146/332	212	157	74	25	7
Zonisamide	167/328	185	128	59	23	7
Percentage without failure (95% CI) Lamotrigine		80 (75, 84)	76 (71, 80)	68 (62, 73)	61 (53, 68)	61 (53, 68)
Percentage without failure (95% CI) Levetiracetam		70 (65, 75)	60 (54, 65)	52 (46, 58)	45 (37, 52)	45 (37, 52)
Percentage without failure (95% CI) Zonisamide		64 (58, 69)	53 (47, 59)	45 (39, 52)	37 (30, 45)	27 (16, 37)
Difference in percentage with failure compared with Lamotrigine (95% CI)						
Levetiracetam		10 (3, 17)	16 (8, 23)	16 (7, 24)	16 (5, 27)	16 (5, 27)
Zonisamide		16 (9, 23)	23 (15, 30)	22 (14, 30)	23 (13, 34)	34 (21, 47)

Table 4. Doses taken by participants aged 12 years and above at treatment withdrawal or last follow up

Reason for withdrawal	Lamotrigine	Levetiracetam	Zonisamide
Inadequate seizure control	n=14	n=16	n=25
First follow-up/Missing[1]	(FF=1)	(FF=1, missing=1)	(FF=1)
Mean mg (s.d.)	267 (152)	2214 (955)	277 (136)
Range mg	75 to 500	500 to 3500	100 to 550
Unacceptable adverse Events	n=34	n=63	n=77
First follow-up/Missing	(FF=16)	(FF=18)	(FF=20, missing=3)
Mean mg (s.d.)	171 (69)	1089 (473)	205 (101)
Range mg	50 to 300	10 to 2500	25 to 500
Other reason for withdrawal	n=17	n=17	n=28
First follow-up/Missing	(FF=6)	(FF=8)	(FF=9, missing=1)
Mean mg (s.d.)	164 (94)	1188 (667)	242 (83)
Range mg	75 to 400	500 to 3000	150 to 400
Remission of seizures	n=7	n=7	n=10
First follow-up/Missing	0	FF=1	FF=1
Mean mg (s.d.)	183 (149)	1029 (221)	200 (61)
Range mg	50 to 500	800 to 1500	100 to 250
Still on randomised drug	n=238	n=188	n=149
Missing	(missing n=11)	(missing n=10)	(missing n=17)
Mean mg (s.d.)	222 (116)	1440 (726)	247 (112)
Range mg	50 to 700	250 to 4000	25 to 600

[1] For patients who had withdrawn from drug at or before first follow-up, no information on the final dose was collected. FF denotes these patients, missing denotes other patients with missing dose information

Web figure 4. Cumulative incidence plots from time to treatment failure competing risks analysis: Lamotrigine versus levetiracetam versus zonisamide.

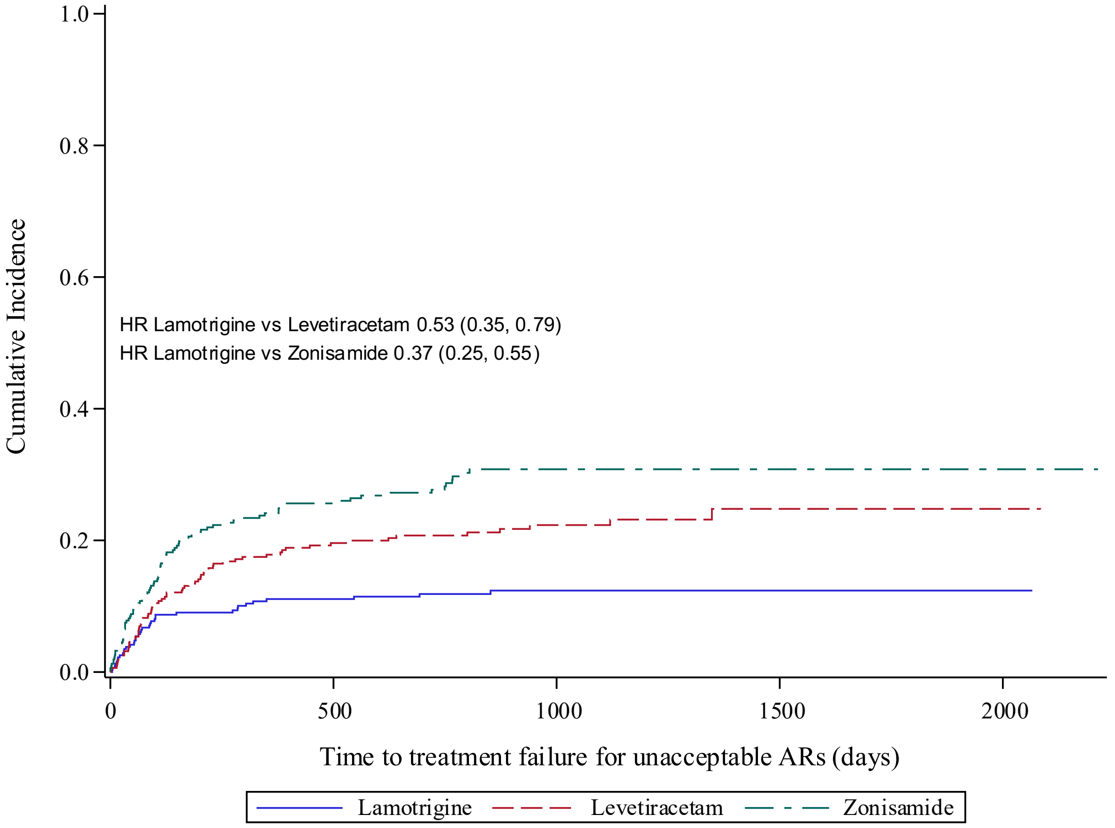

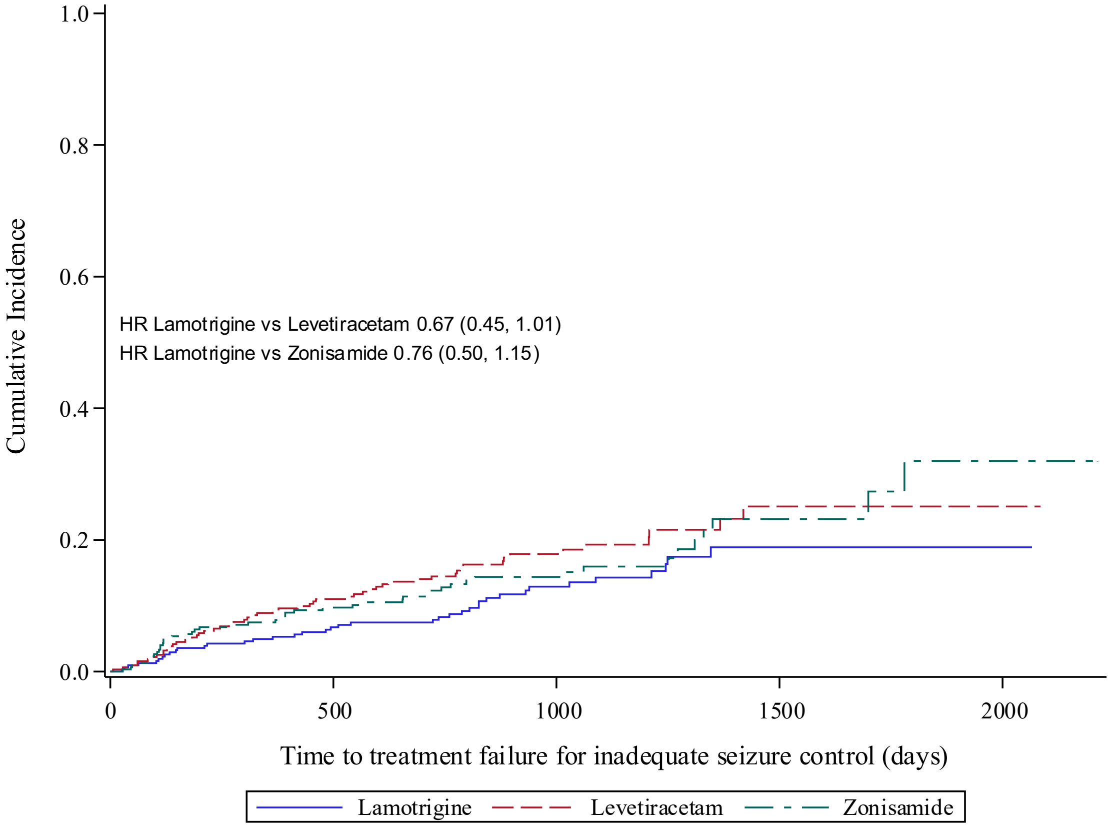

Web table 5. Comparison of the characteristics of those that did and did not return quality of life questionnaires

	No return	Return	Total
Age (years)			
n	497	493	990
Mean (S.D.)	34.2 (18.6)	44.5 (22.3)	39.3 (21.2)
Median (IQR)	32.2 (20.2,45.1)	44.9 (24.8,64.2)	37.7 (22.6,54.5)
Range	5.0 to 88.8	5.0 to 91.9	5.0 to 91.9
Missing	0	0	0
Gender			
n	497	493	990
Male	288 (57.9%)	273 (55.4%)	561 (56.7%)
Female	209 (42.1%)	220 (44.6%)	429 (43.3%)
Learning disability			
n	497	493	990
Yes	28 (5.6%)	17 (3.4%)	45 (4.5%)
No	469 (94.4%)	476 (96.6%)	945 (95.5%)
Neurological deficit			
n	497	493	990
Yes	28 (5.6%)	16 (3.2%)	44 (4.4%)
No	469 (94.4%)	477 (96.8%)	946 (95.6%)
Previous or current neurological disorder			
Stroke/cerebrovascular	21 (4.2%)	26 (5.3%)	47 (4.7%)
Cerebral haemorrhage	10 (2.0%)	4 (0.8%)	14 (1.4%)
Intracranial surgery	12 (2.4%)	8 (1.6%)	20 (2.0%)
Head injury: Post traumatic amnesia>24h or compound depressed fracture	10 (2.0%)	8 (1.6%)	18 (1.8%)
Meningitis/encephalitis	9 (1.8%)	8 (1.6%)	17 (1.7%)
Cortical dysplasia/developmental anomaly	4 (0.8%)	0	4 (0.4%)
Other	29 (5.8%)	40 (8.1%)	69 (7.0%)
History			
Febrile convulsions	27 (5.4%)	17 (3.4%)	44 (4.4%)
Any other acute symptomatic seizures	10 (2.0%)	9 (1.8%)	19 (1.9%)
Family history of epilepsy in primary relatives	71 (14.3%)	36 (7.3%)	107 (10.8%)

NOTE: Return group includes those who were included in any longitudinal analyses – i.e. returned baseline questionnaire and at least one other time point (child, parent or adult)

Web table 6. Quality of life analysis: Adults

QoL variable	Number patients included in analysis	Treatment effect estimate (LTG vs LEV)[1]	95% CI	p-value	Treatment effect estimate (LTG vs ZON)[1]	95% CI	p-value
**Adults**							
Adverse events profile	405	-1.39	-3.14, 0.36	0.118	-0.89	-2.67, 0.89	0.327
Anxiety	406	-1.33	-2.03, -0.64	<0.001	-0.22	-0.93, 0.49	0.544
Depression	406	-1.20	-1.83, -0.56	<0.001	-0.80	-1.45, -0.15	0.015
Mastery	364	0.36	-0.19, 0.91	0.199	0.32	-0.25, 0.89	0.276
Stigma	365	-0.50	-0.96, -0.05	0.031	0.01	-0.46, 0.48	0.967
Impact	362	1.86	0.73, 3.00	0.001	1.82	0.65, 2.99	0.002
Overall QoL	358	-0.52	-0.77, -0.26	<0.001	-0.41	-0.67, -0.15	0.002

[1] Negative treatment effect estimates favour Lamotrigine, with the exception of Mastery and Impact, where positive estimates favour Lamotrigine

Web table 7. Quality of life analysis: children.

QoL variable	Number patients included in analysis	Treatment effect estimate (LTG vs LEV)[1]	95% CI	p-value	Treatment effect estimate (LTG vs ZON)[1]	95% CI	p-value
**Children (self-reported)**							
Attitude to epilepsy	32	-1.40	-17.38, 14.58	0.860	-9.46	-23.79, 4.86	0.189
QoL Physical	31	-0.89	-17.27, 15.50	0.913	-1.01	-16.10, 14.08	0.892
QoL Emotional	31	-8.01	-19.99, 3.97	0.184	-6.31	-17.26, 4.65	0.251
QoL Self esteem	30	-9.54	-25.85, 6.77	0.243	4.97	-10.16, 20.09	0.510
QoL Social	31	-1.86	-12.87, 9.15	0.734	1.87	-8.56, 12.29	0.718
QoL Family	31	-13.82	-29.44, 1.80	0.081	-7.44	-21.84, 6.96	0.302
QoL School	30	-18.75	-32.88, -4.62	0.011	-12.43	-25.35, 0.50	0.059
Impact of epilepsy	7	1.82	-27.06, 30.70	0.888	-4.81	-27.58, 17.95	0.639
**Parent proxy reported**							
QoL Physical	62	-4.22	-13.93, 5.48	0.391	-6.10	-15.49, 3.28	0.201
QoL Emotional	61	0.10	-9.09, 9.29	0.983	0.34	-8.32, 9.00	0.938
QoL Self esteem	60	-5.44	-13.58, 2.70	0.189	-2.39	-10.15, 5.37	0.544
QoL Social	60	-9.45	-18.06, -0.83	0.032	-5.02	-13.11, 3.08	0.222
QoL Family	61	1.28	-7.17, 9.73	0.765	1.36	-6.62, 9.34	0.736
QoL School	61	-8.53	-17.59, 0.52	0.065	-5.17	-13.79, 3.44	0.237

[1] Positive treatment effect estimates favour Lamotrigine

Table 8. Total, unadjusted costs by category of resource use.

	Mean costs (97·5% CR) (£)		
**Item of resource use**	**Lamotrigine**	**Levetiracetam**	**Zonisamide**
Primary & community care	682 (551, 1018)	1303 (981, 2009)	1013 (786, 1631)
Primary care	332 (284, 423)	532 (416, 724)	411 (347, 567)
Community care	350 (228, 646)	771 (489, 1381)	602 (374, 1117)
Secondary care	3025 (2606, 3628)	3263 (2853, 3723)	3882 (3140, 4670)
Admitted patient care	1170 (855, 1631)	1156 (869, 1443)	1663 (1153, 2246)
Outpatient	1519 (1393, 1664)	1705 (1552, 1876)	1784 (1547, 2050)
Accident & emergency	336 (269, 425)	402 (314, 528)	434 (316, 582)
Medicines	356 (294, 475)	508 (412, 665)	515 (423, 668)
Anti-seizure medications	125 (103, 158)	248 (213, 292)	269 (244, 298)
Concomitant medications	231 (175, 348)	260 (172, 403)	246 (161, 390)
Total	4063 (3617, 4842)	5074 (4433, 6049)	5409 (4584, 6658)
